# Precise and economic FIB/SEM for CLEM: with 2 nm voxels through mitosis

**DOI:** 10.1007/s00418-018-1681-x

**Published:** 2018-05-23

**Authors:** Manja Luckner, Gerhard Wanner

**Affiliations:** Department Biology I, Ultrastructural Research, , Ludwig-Maximilians-University Munich, 82152 Planegg-Martinsried, Germany

**Keywords:** FIB/SEM, Golgi, Midzone, Mitosis, Nuclear Envelope

## Abstract

A portfolio is presented documenting economic, high-resolution correlative focused ion beam scanning electron microscopy (FIB/SEM) in routine, comprising: (i) the use of custom-labeled slides and coverslips, (ii) embedding of cells in thin, or ultra-thin resin layers for correlative light and electron microscopy (CLEM) and (iii) the claim to reach the highest resolution possible with FIB/SEM in xyz. Regions of interest (ROIs) defined in light microscope (LM), can be relocated quickly and precisely in SEM. As proof of principle, HeLa cells were investigated in 3D context at all stages of the cell cycle, documenting ultrastructural changes during mitosis: nuclear envelope breakdown and reassembly, Golgi degradation and reconstitution and the formation of the midzone and midbody.

**Electronic supplementary material:**

The online version of this article (10.1007/s00418-018-1681-x) contains supplementary material, which is available to authorized users.

## Introduction

Four ultrastructural techniques are established for 3D-reconstruction of biological specimens: (i) cryo-TEM tomography highest resolution, but limited in section thickness (approx. 500 nm); (ii) serial block face sectioning (3View^®^; large volumes; limited resolution and charging problems; (iii) array tomography (non-destructive; limited resolution in z); and (iv) focused ion beam scanning electron microscopy (FIB/SEM) tomography (larger volumes and highest resolution in z). There is no doubt, that cryo-TEM tomography is the state of the art technique for structural preservation and resolution of sub-cellular structures, however, with severe limitations, when investigating larger volumes in 3D. At present, section thickness is 500 nm at maximum, with a macromolecular resolution of about 3 nm. Comparing resolutions of 3View^®^, array tomography and FIB/SEM-tomography, there are no significant differences in xy (approx. 5 nm), but only possible after metal impregnation. The differences in resolution in z direction are, however, striking: 3View^®^ and array tomography with 20 nm section thickness under best conditions, surpassed by FIB/SEM-tomography by a factor of 10 (Xu et al. 2017). As iso-voxels are necessary for adequate high-resolution 3D-representation in all spatial directions, FIB/SEM is the only technique allowing iso-voxels below 5 nm for large volumes. In daily routine, LSM data sets of entire cells can be recorded within few minutes, providing data for profound statistics, if desired. Corresponding ultrastructural information is not possible with TEM tomography at all, due to volume restrictions. FIB/SEM would offer both, quantitative and high-resolution data sets of entire cells, which can be correlated to LM data. CLEM is still impeded by embedding cells/tissues in resin blocks, due to complicated and time-consuming relocation of target cells, insufficient for statistical investigation.

CLEM could be more efficient by preparing biological samples appropriate for FIB/SEM right from the beginning. As it is crucial to define the coordinates of a target area for re-localization in SEM we developed a variety of slides with coordinates, successively improved for different demands (Schroeder-Reiter et al. 2012). Our aim was to embed cells on slides or cover slips within an ultra-thin resin layer (i) for rapid and precise correlation to LM micrographs and (ii) to allow FIB-milling in any desired direction. Several modified protocols are available using thin embedding, but lack correlation to LM (Belu et al. 2016; Schieber et al. 2017) or involve delicate and critical preparation steps for CLEM (Verkade 2008; Murphy et al. 2011; Rennie et al. 2014; Booth et al. 2016; Lees et al. 2017; Santoro et al. 2017). In a recent book chapter the technical possibilities for various embedding protocols (classical *en bloc* embedding and thin-layer plastification) are presented for live cell imaging with volume scanning electron microscopy (Lucas et al. 2017). Ultra-thin embedding was adapted in our lab to a wide spectrum of biological specimens (from prokaryotes to tissues) and various fixation techniques. Technical improvements for precise and economic CLEM focused on following aspects:

- Conservation of cell topography from LM to SEM.
- Adaption of the thickness of the resin layer to any demand.
- Immediate and precise correlation between LM and SEM.
- Enabling direct access to the target cell to omit a ramp.
- Reduction of the entire milling volume to its minimum, the cell volume.
- Incorporating the slide as an absolute reference for precise alignment of the FIB-stack.
- Including volume rendering for direct 3D visualization at high-resolution.

Mouse C2C12 myoblast cells, stable expressing a fusion of GFP to DNA methyltransferase 1 (GFP-Dnmt1), visible in late S-phase as many looped or toroidal spots (Leonhardt et al. 1992; Schneider et al. 2013), were used for determination of precision of CLEM in a sub-micrometer range. HeLa cells were investigated in detail for ultrastructural changes during the cell cycle to illustrate the enormous potential of this technique, providing new 3D insights in metamorphosis of the Golgi, nuclear envelope breakdown and reconstitution, formation of the midzone and midbody, based on high-resolution 3D FIB/SEM data sets. The economy of FIB/SEM was improved by optimizing all technical parameters to achieve a voxel-size of 2 × 2 × 2 nm over hundreds of sections.

## Materials and methods

### Cell culture

**Fig. 1:**
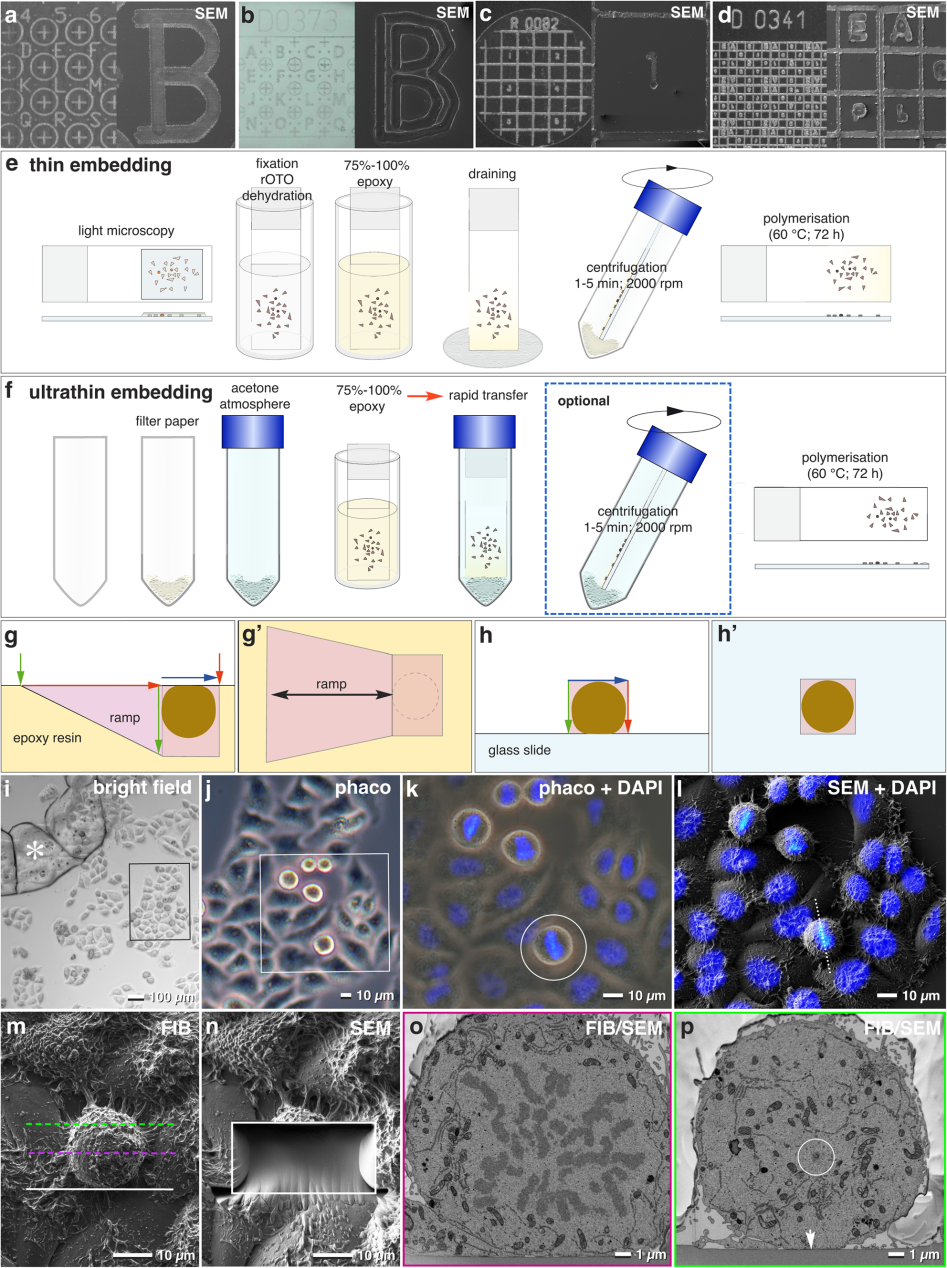
Ultra-Thin Embedding of Cells: Precise and Economic CLEM. **a**–**d** Close-up photographs of laser marked slides and coverslips with different coordinates and label properties and corresponding SEM micrographs. Labels are seen as indentations in SEM, best suitable for ultra-thin embedding (**a, b**). For thin embedding, raised labels are of advantage for better visualization in SEM (**c, d**). **e, f** Workflow for thin (**e**) and ultra-thin (**f**) embedding. For thin embedding, a simple draining of epoxy resin in concentrations from 75 to 100% can be adequate for larger cells/objects. After centrifugation, the epoxy layer is significantly reduced, but a slight gradient in thickness at the lower part of the slide is typical (e). For ultra-thin embedding, a filter paper, saturated with acetone, is inserted at the bottom of a Falcon^®^ tube to provide an acetone atmosphere, which prohibits increase of resin viscosity, occurring within seconds to few minutes. Simple draining in an upright position results in a very thin resin layer. After centrifugation, the resin layer is extremely thin, surface details of cells appear to be uncovered (**f**). **g, h** Comparison of FIB/SEM milling of a conventionally embedded cell within a resin block, which requires a deep ramp (**g** = side view; **g’** = top view) or ultra-thin embedded on a laser marked slide (**h**). As a deep ramp is needless, milling and block face imaging can start directly at the cell (**h** = side view; h’ = top view). The volume that has to be milled (pink) for an entire data set of a cell is reduced to 10% (**h, h’**). i Bright field light micrograph of HeLa cells, grown on slide with laser marks (asterisk) serving as coordinates to retrieve target cells in the SEM (framed area). Scale bar 100 µm.** j** Phase contrast micrograph of the target region from (**i**). Dividing cells are spherical and appear bright (framed area). Scale bar 10 µm. **k** Merged DAPI fluorescence and phase contrast micrographs (framed area of **j**) shows mitotic stages and a target cell (circle) with upright orientation of the metaphase plate. Scale bar 10 µm. **l** Merged SEM and DAPI micrographs of the target area. After ultra-thin embedding in epoxy resin, the target cell is precisely relocated. The axis of the metaphase plate is in upright position (dotted line). Scale bar 10 µm. **m** SEM micrograph of the target cell (FIB image), oriented for FIB/SEM milling parallel to the metaphase plate. White line = starting position for milling; magenta line = position of metaphase plate; green line = expected position of the distal centrosome. Scale bar 10 µm. **n** SEM micrograph of the target area shown in (**m**) after FIB/SEM milling (framed area). Image acquisition started direct in front of the cell and stopped just after reaching the desired depth. Scale bar 10 µm. **o, p** Selected SEM micrographs from the FIB/SEM-tomogram (magenta and green dotted line in (**m**), illustrating the position of the images shown in (**o**) and (**p**). 1100 micrographs cover the entire metaphase plate including the centrosomes (circle). Arrow indicates the reference line (slide) for precise alignment. Scale bar 1 µm

HeLa Kyoto and mouse C2C12 myoblast cells were kindly provided by Prof. Dr. Heinrich Leonhardt. Cells were cultured in DMEM (Thermo Fisher Scientific) + 10% FBS (GIBCO) and Gentamicin (5 µg/ml) (Thermo Fisher Scientific). Laser marked slides or coverslips (Fig. 1a–d) were placed in a dish and cells were grown in an incubator at 37 °C, 5% CO_2_ in a water vapor saturated atmosphere, until an appropriate density on the slides was reached (30–50%).

### LM of HeLa cells and mouse C2C12 myoblast cells

Slides/coverslips were rinsed with PBS (Thermo Fisher Scientific) and immediately fixed with 2.5% glutardialdehyde (Science Services GmbH, München) in 75 mM cacodylate (Sigma–Aldrich), 75 mM NaCl, 2 mM MgCl_2_ for 30 min, followed by 3 washing steps in cacodylate buffer. Cells were stained with DAPI, sealed with a coverslip and Fixogum (Marabu GmbH & Co. KG, Tamm, Germany) to prevent drying during LM investigation. ROIs were marked on a template, with the same coordinates (Fig. 1a–d). For documentation 2–3 different magnifications (Objective: 5x, 10x,  40x) were sufficient to retrieve ROIs in SEM. Depending on specimen properties, bright field, phase contrast, DIC were used. Different areas were documented, as (i) there can be a loss of few cells and/or damage during handling; (ii) some cells may show insufficient fixation quality or contrast in SEM among neighboring cell, adequate in structure and contrast. For correlative CLSM of mouse C2C12 myoblast cells, overview images were acquired in “tile scan” mode with a LSM 780 with a Plan-Apochromat 40x/1.3 oil DIC M27 objective (Zeiss, Germany) to select cells in the desired stage, identified by the characteristic signal of GFP-Dnmt1. Emission of green fluorescent protein (GFP) and DAPI was collected using standard filter sets for GFP (486–564 nm) and DAPI (403–473 nm). Confocal z-stacks (113.27 × 113.27 × 15.86 µm) were recorded with an image pixel size of 78 nm in xy and 260 nm in z.

### EM preparation

After removal of Fixogum and cover slip, cells were post-fixed (customized rOTO-protocol) with 1% OsO_4_ and 1% K_4_Fe(CN)_6_ in cacodylate buffer for 30 min, washed 3 times in ddH_2_O, incubated with 1% thiocarbohydrazide in ddH_2_O for 30 min, washed with ddH_2_O 3 times, followed by post-fixation with 1% OsO_4_ in ddH_2_O for 30 min. The samples were rinsed 3 times with ddH_2_O and dehydrated in a graded series of acetone (10%, 20%, 40%, 60%, 80%, 100%), containing a 1% uranyl acetate step in 20% acetone for 30 min, infiltrated and embedded on the glass slide (Fig. 1e, f).

#### Thin embedding

Cell were infiltrated with 1:1 Hard-Plus Resin-812 in acetone for 15 min, 2:1 for 30 min, 75–100% Hard-Plus Resin-812 for 30 min at RT. Excessive resin was removed by centrifugation (2 min; 1000 rpm) (Fig. 1e).

#### Ultra-thin embedding

Cells were infiltrated with 1:1 Hard-Plus Resin-812 in acetone for 15 min, 2:1 (resin/acetone) for 30 min and 3:1 (resin/acetone) for 30 min. A filter paper, completely soaked with acetone, was placed at bottom of a Falcon^®^ tube to provide an acetone saturated atmosphere. A polypropylene cap was placed on top of the filter paper to avoid direct contact with the slide. The slide was placed upright into the Falcon^®^ tube, allowing the excessive resin to drain into filter paper at the bottom of the Falcon^®^ tube for 10–30 min. If necessary, an additional centrifugation can be added (2 min, 1000 rpm) (Fig. 1f).

The samples were polymerized for 72 h at 60 °C. The size of the glass slides was reduced to appropriate size by fracturing with aid of a diamond pen. The specimens were mounted on an aluminum stubs with colloidal silver.

#### Conductive coating

Platinum is the metal coating commonly used for scanning electron microscopy (SEM). Due to its high back-scattered electron (BSE) yield, platinum coating prohibits material contrast of sub-surface structures. Carbon coating allows both, high-resolution BSE and topographic secondary electron (SE) images, despite the lower SE-yield of carbon—which is in practice compensated—as for BSE/energy selective back-scattered electron (EsB) imaging, larger apertures (60 µm) and high current mode are preferentially used. A carbon coating of 15 nm thickness for FIB/SEM-tomography is preferred to any sputter coating with heavy metals for the following reasons: metal grains deposited by sputtering, separate under prolonged exposition to the electron beam and lead to charging. The high yield of SE favored by heavy metal coating, images the upper few nm of the surface. The surface of the specimen itself is only part of the information needed for correlation to LM micrographs. Together with the subsurface information of the BSE signal, a very precise correlation of LM data sets is given.

### High-resolution SEM

The most important SEM parameters should be picked out to illustrate the potential for CLEM. For the first step, simple correlation of size and shape of a specimen in LM and SEM, working distance has to be large (10 mm) and kV high (5 kV) to ensure a sufficient low magnification with acceptable low geometrical distortion in SEM. Although surface details are best monitored at 1 kV with the inlens SE detector, correlation with LM micrographs needs as much as possible depth information. This information is gathered by the EsB detector (1–5 kV) or at higher kV (5–30 kV) with the 4-quadrant back-scattered electron detector (QBSD) detector. Thin layers of resin become transparent and laser marks are clearly visible. When using BSE signals, a larger aperture is necessary for a sufficient signal to noise ratio, which does not influence resolution a low and moderate kV. The high current mode is of benefit if the depth of focus is of importance: high current mode increases the active probe current by a stronger activation of the condenser lens. The resulting smaller angle of convergence increases the depth of field. LM and SEM images perfectly match after merging. Only minor corrections, as rotation and some linear scaling are sufficient for a fast and precise correlation.

### High-resolution FIB/SEM

HeLa and mouse C2C12 myoblast cells were imaged in an Auriga 40 FIB/SEM workstation operating under SmartSEM^®^ (Carl Zeiss Microscopy GmbH, Oberkochen, Germany) or Atlas 3D (Fibics incorporated, Ottawa, Canada). FIB/SEM milling was started right in front of the cell. Ion beam currents (dependent on the stability of the resin) of 50 pA to 10 nA were used. Due to ultra-thin embedding, milling of a trench is needless and direct excess to the target structure is given (compare Fig. 1g, h, m, n). Dependent on the desired resolution, image pixel sizes between 2 nm and 10 nm in x/y were chosen. Milling rate was set to 2 nm, which allows the adjustment of the z-resolution in 2 nm steps at any time during the FIB/SEM run. Due to metallic rOTO impregnation of the tissue, carbon coating, conducting with colloidal silver and, if necessary, Pt-deposition upon the ROI, charging was completely avoided. The rOTO impregnation provides a strong material contrast; therefore, shorter exposure times down to 17 s/image (3072 × 2048 pixel) could be achieved.

### 3D-reconstruction

The datasets were aligned using Amira™ (Thermo Fisher Scientific, USA) with the module *align slices*. The image stacks, either from CLSM or FIB/SEM were segmented and reconstructed in Amira™ or processed with an direct volume rendering algorithm (volren) for immediate visualization. For correlative microscopy of Dnmt1, landmarks, as characteristic surface details of the nucleus, were used as reference points to correlate CLSM and FIB/SEM data sets. With the *Landmark Surface Wrap* option, the “DAPI nucleus” was adapted to the “FIB/SEM nucleus” by aligning the previous set reference points and further optimized by manual transformations.

## Results

### Locating target cells in routine

Changes in the 3D ultrastructure of HeLa cells were studied in the context of developmental and/or functional aspects. The entire EM preparation of cells was performed on customized laser marked coverslips/slides to track the position from LM through SEM investigation (Fig. 1). Several coverslips/slides with different coordinate systems varying in size and type, either engraved or elevated by a sinter process (Fig. 1a–d) were produced for either thin- or ultra-thin embedding (Fig. 1e, f). After ultra-thin embedding, the appearance of the cells remains unaltered from LM to SEM, enabling a fast retrieval of target cells (Fig. 1i–l). The thickness of epoxy layers can be adjusted using 75–100% epoxy/acetone mixtures as final concentrations (Fig. 1e, f) and adapted by draining and centrifugation or by an acetone saturated chamber and an optional centrifugation step (Fig. 1f). Excellent structural preservation enables easy recognition and relocation of HeLa cells in LM and SEM (Fig. 1i–l). Merging light micrographs with SEM micrographs, the target cells fit perfectly (Fig. 1l). If thin resin layers obscure laser markings, increasing the accelerating voltage (3–5 kV) enhances the overall material contrast. For smooth cell surfaces, ultra-thin epoxy layers are optimal. If the cell surface is extremely structured, a thin embedding is advantageous to avoid curtaining and therefore slides with elevated coordinates, which poke out and are not covered by the epoxy resin, are favored (Fig. 1d).

### Economic FIB-SEM milling

FIB/SEM milling of a target cell is very efficient, as the cells remain on the substrate and thin embedding enables a fast re-localization of selected ROIs (Fig. 1m, n). Cells, classical embedded in a resin block, a ramp has to be milled, to get access to the target ROI (Fig. 1g, g’). With ultra-thin embedding, a ramp is needless, and only the actual volume of the ROI is ablated without redeposition effects (compare Fig. 1g with h). The required ablation volume for a single cell within a resin block is ten times larger, compared to cells after ultra-thin embedding (Fig. 1g, h). The position and orientation of sub-cellular structures in top view (e.g., metaphase plate) can be determined quickly with precision in the micrometer range (Fig. 1k–m). With ion beam currents of 2–5 nA, an HeLa cell can be milled to its center within 5–20 min. An image stack of a centrosomal region with several hundred micrographs can be recorded with 2 nm iso-voxels within a few hours (Fig. S1; Movie S1).

### Fast imaging and precise alignment

During FIB-milling, the cross section of the glass slide serves as absolute reference for precise alignment (Fig. 1h, o, p). Demonstrating the necessity of a reference for immaculate alignment, an image stack including the glass slide as base line (Fig. S2a) was cropped (Fig. S2b), aligned separately and compared in yz view, illustrating striking differences in shape and position of cellular structures, especially if they are elaborate and filigree as e.g. ER (Fig. S2a’, S2b’). For FIB/SEM, the signal of the in-lens EsB detector is standard. Approaching the resolution limit, applying the inlens SE signal has several advantages: better resolution, better signal to noise ratio and much shorter exposure times (Villinger et al. 2012). With an increased heavy metal impregnation (rOTO), high-resolution images (3072 × 2048 pixel) can be taken within 17–23 s, compared to the EsB signal with 30–50 s (Fig. 1o, p). However, finest curtaining is immediately visible in the inlens SE image (not shown).

### CLEM of Dnmt1 In mouse C2C12 myoblast cells

**Fig. 2:**
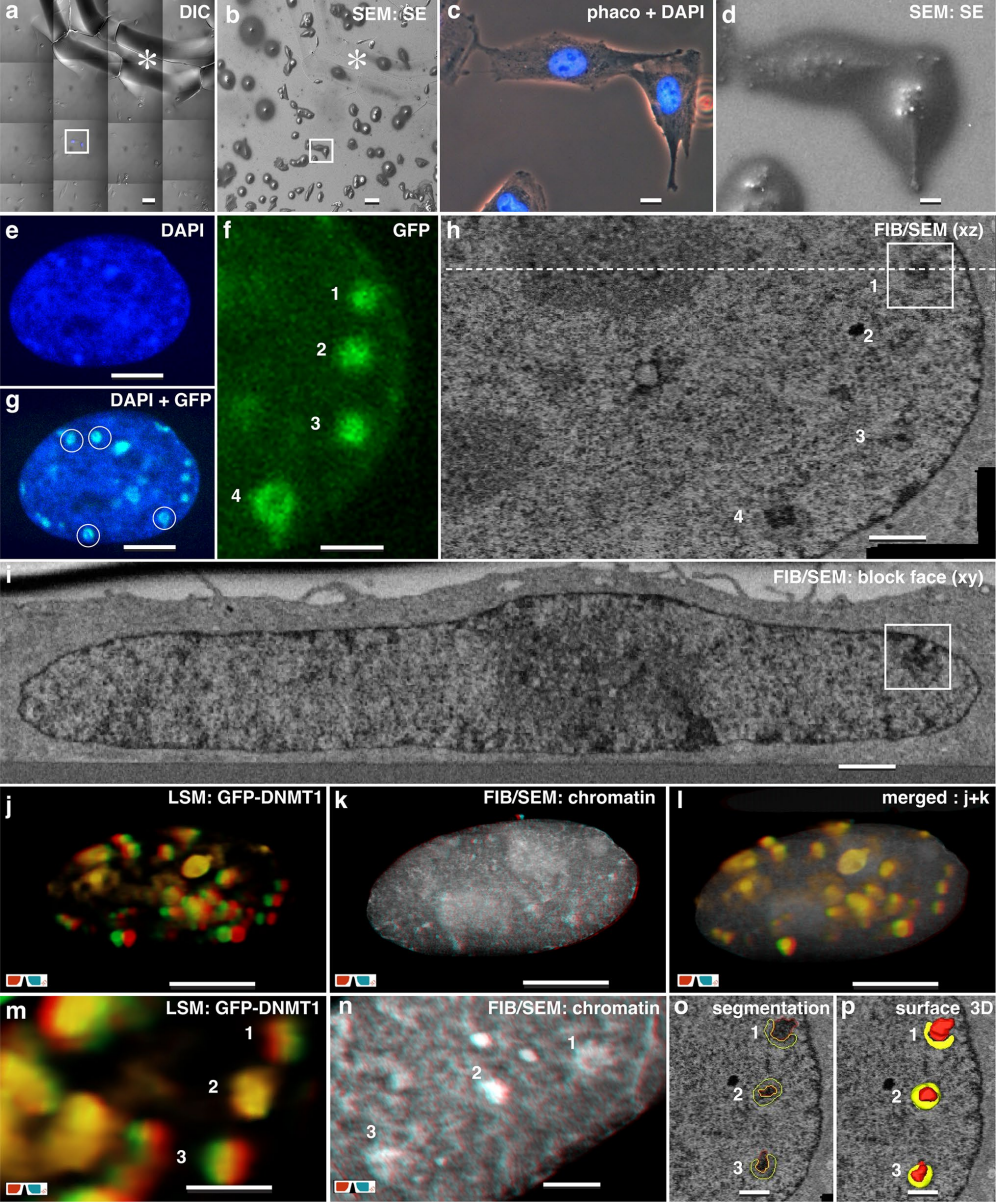
CLEM of Dnmt1 in mouse C2C12 myoblast Cells. **a** Light micrograph (DIC) of mouse C2C12 myoblast cells, stable expressing GFP-Dnm1, grown on a laser marked coverslip and counter stained with DAPI, imaged in tile scan mode. Scale bar 100 µm. **b** Scanning electron micrograph of the target area from **a**. The coordinates of the coverslip allow precise re-localization in SEM, as they are visible after thin embedding (asterisk). Scale bar 100 µm. **c** Light micrograph of target cell (phase contrast) merged with DAPI signal. Scale bar 10 µm. **d** Scanning electron micrograph of the target cell (SE image). Scale bar 10 µm. **e**–**g** Maximum intensity projection of confocal image stacks of DAPI (**e**) GFP-Dnmt1 (**f**) and merged (**g**) of the target cell from (**c, d**). Scale bar in **e** and **g** 10 µm; in **f** 5 µm. **h** FIB/SEM micrograph of the target cell in top view (xz). The section plane shows 4 chromocenters with same arrangement as GFP-signals from (**f**). The dotted line marks the position of the block face image presented in (**i**). Scale bar 1 µm. **i** FIB/SEM micrograph of the target cell in front view (xy). The section plane shows the region of the chromocenter 1 (frame). Scale bar 1 µm. **j**–**l** 3D visualization by volume rendering of GFP-Dnmt1 signal (**j**) FIB/SEM nucleus (**k**) and correlation of both volumes (**l**). Scale bar 5 µm. **m**–**n** Detail of 3 prominent GFP spots in 3D (**m**) and corresponding to their ultrastructure, based on the FIB/SEM volume **n**. Scale bar in m 1 µm; in n 500 nm. **o**–**q** FIB/SEM micrograph of 3 chromocenters, characterized by an electron dense core surrounded by a less electron dense hem, identified and segmented based on their electron density (**o**) reconstructed in 3D (**p**) and merged with the GFP signal (**q**) demonstrating the co-localization. Scale bar in **o** and **p** 500 nm

The precision of the CLEM workflow is demonstrated by localization of the DNA methyltransferase 1 (Dnmt1) during late S-phase in mouse C2C12 myoblast cells. Dnmt1 is enriched in replication foci at chromocenters, when DNA of peri-centromeric heterochromatin (pHC) is replicated (Schneider et al. 2013). From target cells, grown on labeled slides, confocal z-stacks were recorded. Cells can be easily relocalized after thin embedding by the coordinates and similar appearance in phase contrast and SEM (Fig. 2a–d). The data set from FIB/SEM-tomography was examined in xz-view to facilitate direct comparison to CLSM optical sections (Fig. 2e–h). The GFP-Dnmt1 signal (Fig. 2g, f) correlates with characteristic ultrastructural details: electron dense regions of heterochromatin, surrounded by a less electron dense hem, distributed within the nuclear matrix (Fig. 2h). In multi-planar mode (Amira™), the ROI can be visualized in any desired direction with high-resolution (Fig. 2i, h). 3D reconstructions of the confocal image-stacks were correlated to the FIB/SEM volume to localize and identify the ultrastructure of Dnmt1. As the LM and FIB/SEM data sets are different, both, in resolution and orientation of the axis of their stacks, their surfaces had to be aligned. Due to the lower resolution, the LM reconstruction was adapted to the high-resolution FIB/SEM data. The volume of the nucleus (based on the DAPI signal) was scaled to the 3D coordinates of the nuclear envelope derived from FIB/SEM-tomography (Fig. 2j–n). Two different modes for visualization can be used: volume rendering (based on threshold of grey levels), which is very objective but limited, if very large volumes have to be visualized, as resolution decreases with depth (Fig. 2n). Manual segmentation is time consuming, often subjective, but with the advantage of separating and visualization of several different structural details (Fig. 2o, p). 3D FIB/SEM reconstructions reveal the electron dense regions as a core, sheathed in a less electron dense structure, either completely or in part enwrapping the core (Fig. 2o, p). Dnmt1 rather corresponds to the hem, than the core. GFP-Dnmt1 signals can be generally assigned to similar organization of the chromatin varying from 0.7 µm to 1.5 µm in diameter.

## Ultrastructural changes during mitosis

### Shuttling between nuclear envelope (NE) and ER

**Fig. 3:**
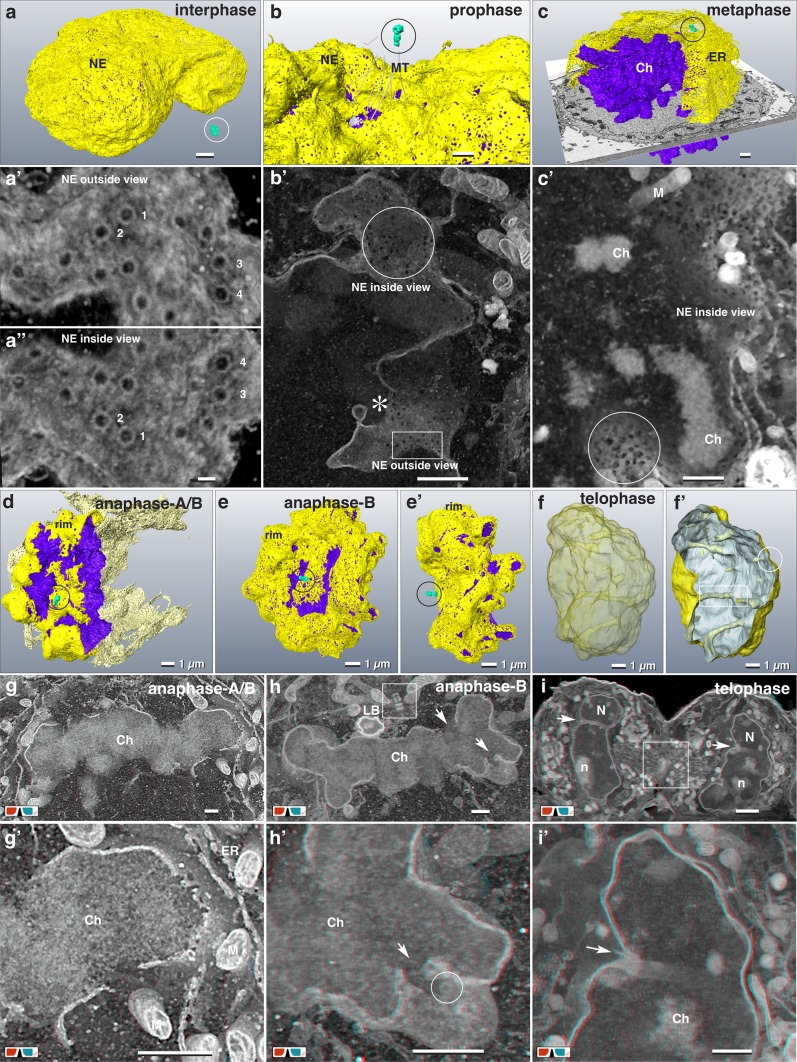
Nuclear envelope breakdown and reconstitution. **a**–**c** 3D-reconstruction (**a**–**c**) and volume rendering (**a’**–**c’**) of the nuclear envelope (NE; yellow) and chromatin (Ch; purple) of HeLa cells in inter- (**a**), pro- (**b**) and metaphase (**c**) with their centrosomes (turquoise). **a, a’, a’’** The nuclear pores are evenly distributed across the NE in interphase (**a**). Volume rendering of the NE, in view from the cytoplasmic side (**a’**) and from the nuclear matrix (**a’’**). High-resolution (2 nm iso-voxel) of the outer and corresponding inner nuclear pore complexes with their subunits (1–4). Scale bar in **a** 1 µm; in **a’** and **a’’** 100 nm. **b** NE in prophase with several openings (asterisk), towards the centrosome. MTs start to enter the NE. Scale bar 1 µm. **b’** With onset of NE degradation in prophase, the NE opens, facing the centrosome (asterisk). The regularly arranged NPs (rectangle) with their NPCs disappear, leaving holes of irregular sizes and shapes (circle) unevenly distributed over the NE. Scale bar 500 nm. **c** The metaphase NE transforms to a fenestrated ER-cage surrounding the condensed chromosomes (Ch), open at the poles, where the centrioles (circle) are located. Scale bar 1 µm. **c’** Volume rendering of a metaphase cell illustrates the fenestrated ER-cage without NPCs (circle), surrounding the condensed chromosomes (Ch). Typically, some mitochondria (M) enter the (former) nuclear matrix. Scale bar 1 µm. **d** 3D-reconstruction of the NE in anaphase. ER-sheets (yellow) encompass the chromosomes (purple) starting at the rim and the proximal side of the chromosome mass. The ER cage (derived from NE breakdown) is still preserved (light yellow). See Movie S2. Scale bar 1 µm. **e** During anaphase-B, ER-sheets creeping over the condensed chromatin, tightly covering the chromosome arms, still presenting their typical anaphase shape (**e**’). Scale bar 1 µm. **f, f**’ 3D-reconstruction of a telophase NE with several nuclear invaginations (circle) and tunnels (rectangle). Scale bar 1 µm. **g, g’** Anaglyph images of anaphase-A/B from (**d**) show the discontinuous covering of the chromatin (facing the centrosome) by ER-sheets. NE formation at the chromatin, distal to the centrosome, is retarded. **g’** Higher magnification form (**g**), an ER-sheet in transition from fenestrated ER to attachment to the chromatin, forming the NE. Scale bar 1 µm. **h, h**’ Anaglyph images of anaphase-B (from **e**) demonstrating the formation of nuclear tunnels by attachment of ER-sheets and tubular ER between neighboring chromosome arms (arrows). Typically, lipid bodies (LB) and mitochondria are located within the region of the spindle apparatus. Square: centrioles; circle: nuclear pore with typical pore complex. Scale bar 1 µm. **i, i**’ Anaglyph images of telophase from (**f**). Nuclei (N) of telophase cells, with almost decondensed chromosomes and formation of nucleoli (**n**). Daughter cells are still connected via midbody (framed area). Typically, several nuclear tunnels and invaginations are present in telophase (arrows). Scale bar 1 µm

3D reconstructions of interphase nuclei reveal their varying shapes, including invaginations and nuclear tunnels. Nuclear pores (NP) are discernible as ring structures (± 120 nm in diameter), homogeneously distributed (Fig. 3a, a’, a’’). Examining image stacks through the NE, the outer nuclear ring complex with its subunits is visible first (Fig. 3a’), followed by a circular opening in the NE and the inner nuclear ring complex (Fig. 3a’’). In prophase, the NE opens at several positions toward both centrosomes, giving access to microtubules (MTs) to enter the nucleus (Fig. 3b, b’). NPs lose their ring complex, either in part or complete and their diameter is variable (Fig. 3b’). Concurrently, the NE becomes fenestrated and the distribution of the (former) NPs becomes inhomogeneous (Fig. 3b’). During metaphase, the NE is transformed into an ER-cage encompassing the chromosomes (Fig. 3c, c’, a). 3D reconstruction shows a fenestrated ER-cage, open at two ends toward the centrosomes (Fig. 3c). The fenestration of the NE is best illustrated by volume rendering (*volren*) (Fig. 3c’). NPs were not detected within the ER-cage. With mitotic progression, the cage stretches, but continues to encompass the chromosomes (Fig. 3c). In anaphase, the chromosomes form a disc-shaped mass with a rim region, a proximal side facing the spindle pole and a distal side facing the cell interior (Fig. 3d). With anaphase progression, a striking contact of ER-sheets with the surface of the chromosome mass at the rim is observed (Fig. 3d, e, g, h; Movie S2). In transition from fenestrated ER to a reassembled NE, smaller openings (diameter: ±125 nm) are locally concentrated. NPs with nuclear pore complexes (NPCs) become visible in anaphase when ER-sheets are encompassing the chromosomes (Fig. 3h’). In anaphase-B, the NE outlines the chromosome shapes of the daughter nuclei (Fig. 3e, e’, h, h’). Interruptions in ER-sheets between neighboring chromosomes indicate the origin of nuclear tunnels (Fig. 3h, h’). In telophase, the NE is completely reconstituted with randomly distributed NPs. Nuclear invaginations and tunnels are visible, varying in number (up to 5 or even more) (Fig. 3f, f’, i, i’). Within the nuclear tunnels, strands of ER and bundles of MTs are frequently present.

### Formation of midzone and midbody

**Fig. 4:**
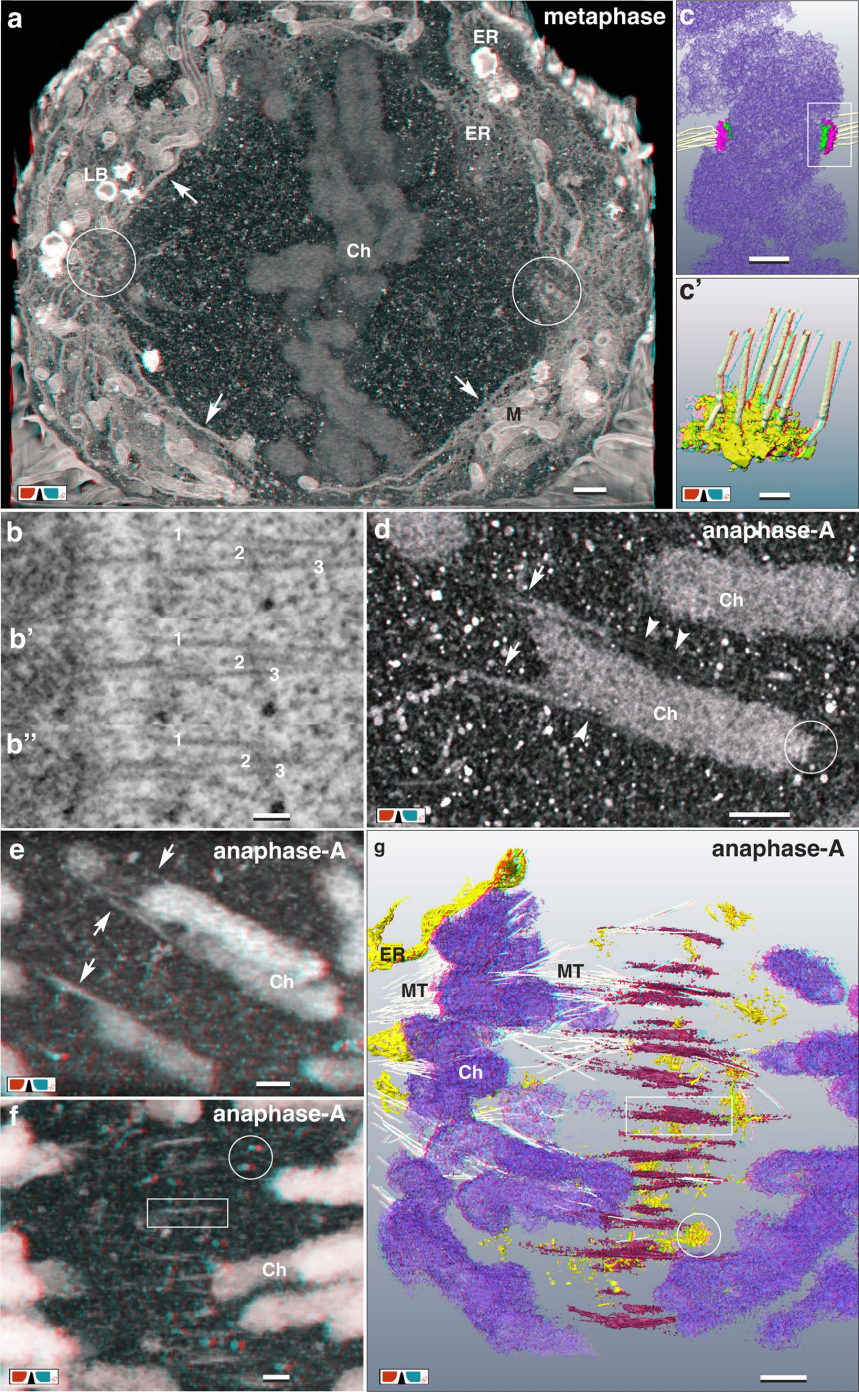
From meta- to anaphase: kinetochores and midzone formation. **a** Chromosomes (Ch) of a metaphase plate surrounded by a fenestrated ER-cage (arrows). Several layers of ER-sheets at the spindle poles are in contact to the centrosomes (circles), probably acting as an anchor. Scale bar 1 µm. **b, b’, b’’** Due to an ablation rate of only 6 nm/section, single MTs, attached to the kinetochore are visible on several consecutive FIB/SEM micrographs. Scale bar 100 nm. **c, c’** 3D-reconstruction of sister kinetochores with their disc-like structure of the larger outer kinetochore (pink) and the smaller inner kinetochore (green). 6–7 MT are attached to the kinetochore. **c’** = detail of **c**. Scale bar in **c** 500 nm; in **c’** 100 nm. **d** Anaglyph image of microtubules (arrowheads) passing the chromosomes flanks, acting as central spindle MTs. Electron dense strands (arrows) seem to be pulled out from the ends of the chromosomes by MTs. Circle marks the kinetochore. Scale bar 500 nm. **e** Anaglyph image of strands of chromatin (arrows), in contact to MTs, emanate laterally from the chromosome arms in anaphase-A. Scale bar 500 nm. **f** Anaglyph image (volume rendering at low resolution for high depth information) of clamp-like structures are arranged midway between the dividing chromosomes (rectangle) in anaphase-A. Characteristic for the midzone is the presence of numerous, electron dense single or aggregated vesicular–tubular clusters (circle). Scale bar 500 nm. **g** Anaglyph image of 3D-reconstruction of chromosomes and midzone in anaphase-A. Clamps (red) are up to 2 µm long and bundle MTs (for clarity only a few are labeled). Halfway between the separating chromosomes (purple), they are arranged in a plane. Numerous vesicular-tubular clusters (circle) are located within the midzone. Scale bar 1 µm

**Fig. 5:**
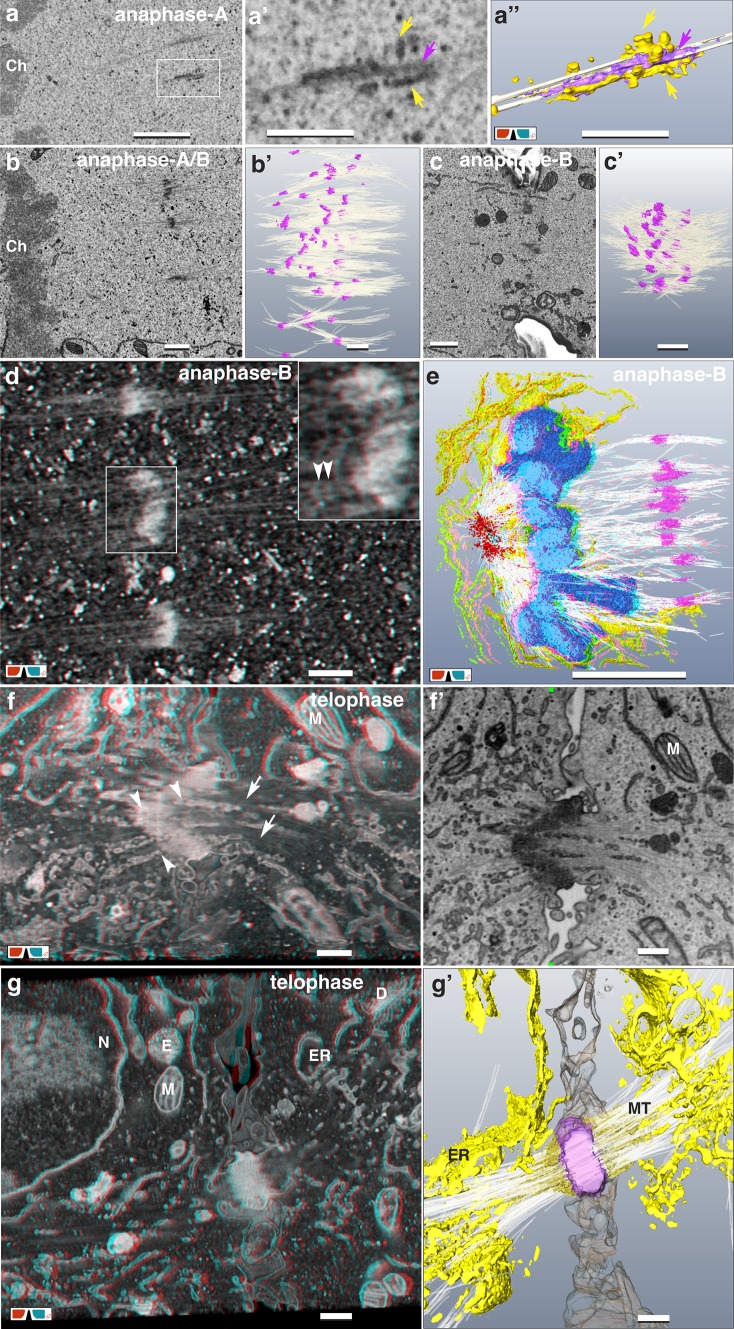
From Midzone to Midbody. **a, a’, a’’** Clamps are built up of single spindle like units (**a**), which bundle 2–3 MTs (**a’, a’’**). At higher magnification, two different zones can be distinguished (**a’, a’’**): a matrix (pink), bundling MTs and attached VTCs (yellow). Scale bar in **a** 1 µm; **a’** and **a’’** 500 nm. **b, b’** With anaphase progression, clamps become more electron dense, begin to cluster and bundle several MTs. Scale bar 1 µm. **c, c’** Clamps in anaphase-B increase in size, simultaneously decreasing in number, by intertwining. Scale bar 1 µm. **d** Anaglyph image of clamps of the midzone in anaphase, discernible as electron dense structures, which are formed by aggregation of several spindle units, bundling numerous MTs. Inset: Next to the clamps, electron dense granular structures, interconnect single MTs (arrowheads). Scale bar 500 nm. **e** Anaglyph image of 3D-reconstruction of a midzone in anaphase-B. Clamps (pink) aggregate, bundling numerous MTs. Nuclear envelope reconstitution starts by attachment of ER-sheets and tubular ER (yellow), to the condensed chromosomes (blue). Scale bar 5 µm. **f, f’** Anaglyph image of midbody in early stage of its formation during telophase. Bundles of MTs permeate the midbody (arrows); tubules and vesicles fuse with the midbody (arrowheads). Scale bar 500 nm. **g, g’** With progression of cytokinesis, the midbody (pink), visible as compact electron dense structure, is located between dividing daughter cells. Microtubules are still present, passing through the midbody and ending diffuse in each daughter cell. (D = dictyosomes; E = endosomes; ER = endoplasmic reticulum; M = mitochondrion; N = nucleus). Scale bar 500 nm

With high-resolution FIB/SEM, kinetochores (KC), characterized by their tripartite appearance, are documented on ± 40 consecutive longitudinal sections (Fig. 4b–b’’ and Fig. S3). Their diameters vary between 200–450 nm (Fig. 4b–d). Distal to the chromatin, a larger disk is located (outer KC), followed by a diffuse layer, adjacent to a second, slightly smaller disc (inner KC) attached to or embedded in the chromatin (Fig. 4b, c). Due to the high-resolution in xyz (2 nm iso-voxel) individual MTs, discerned as two parallel lines in longitudinal sections with a diameter of approx. 25 nm, can be tracked over several sections (Fig. 4b, b’, b’’ and Fig. S3). During anaphase, MTs of the central spindle are attached in parallel to the flanks of the chromosome arms (Fig. 4d). With onset of anaphase, long electron dense strands (0.5-2 µm) are observed, either located halfway between the separating chromatids or emanating from the telomeric regions of the chromosomes (Fig. 4d–f), always attached to microtubules (Fig. 4d–g). Structurally, they are similar to chromatin (Fig. 4d, e). With progression to anaphase-B the midzone is formed: single, electron dense, spindle like structures (Fig. 5a) or aggregates, which we designate as “clamps” (Fig. 5b–d), with a length of up to 2 µm, are arranged in a plane (= midzone) (Fig. 5b–e; Movie S2). Single spindles bundle 2–4 MTs (Fig. 5a); larger clamps bundle up to 25 MTs (Fig. 5–e). With increasing aggregation, the clamps reduce in length to ± 0.6 µm (compare Fig. 5a, d). In later stages they form a compact structure, the midbody (Fig. 5f, g; Fig. S4b). With onset of cytokinesis, densely packed MTs passing the midbody are still visible (Fig. 5f, g; Fig. S4a), ending as bundles in the daughter cells (Fig. 5f, g). Vesicles, vesicular–tubular clusters (VTCs) and tubular ER are located between the MT-bundles, till to the center of the midbody (Fig. 5f, g). A compact midbody can be already formed, even though the envelope of the telophase nuclei has not been reconstituted for each nucleus separately. In this case nuclear bridges are crossing the midbody (Fig. S4). From telophase to cytokinesis the daughter cells move apart, while forming elongated tubular structures (Fig. S4b, S4c), connected to the midbody, which remains in its position. Cytokinesis is characterized by separation of the midbody from the daughter cells (abscission). The tubules exhibit a segmentation by membrane adhering substructures (Fig. S4c), forming sort of short helices similar to structures, described as cortical filaments in the constriction zone of HeLa cells with TEM-tomography (Guizetti et al. 2011; Fededa and Gerlich 2012) and with soft X-ray cryo-tomography (Sherman et al. 2016).

### The Golgi ribbon is not a ribbon

**Fig. 6:**
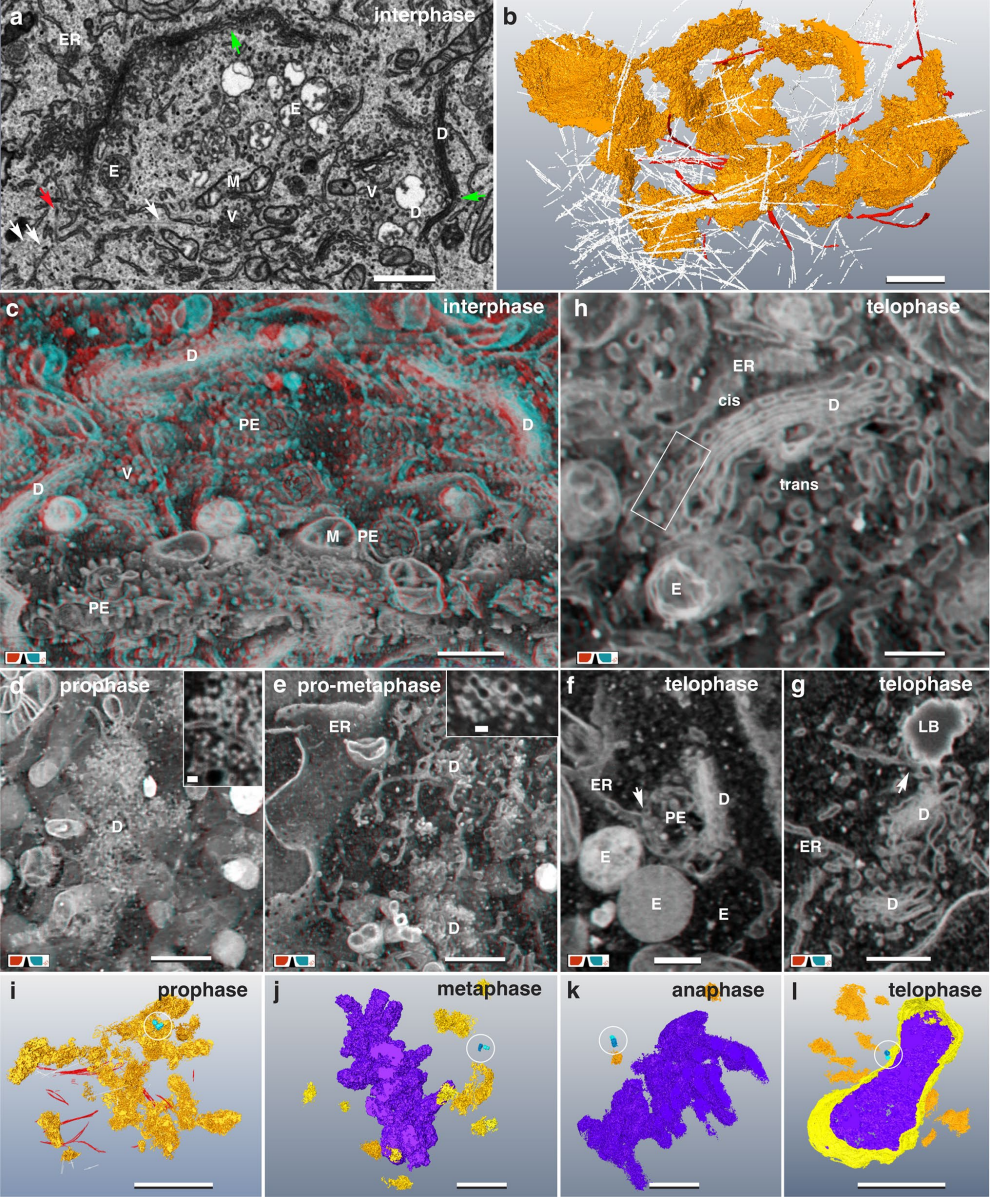
Golgi disintegration and reassembling. **a** SEM micrograph of an interphase Golgi apparatus. Single dictyosomes (D) are interconnected by shared single cisternae (green arrows). Abundant Golgi vesicles (V), primary endosomes (PE) and mitochondria (M) are located in between. MT (white arrows) and actin filaments (red arrow) interlace the Golgi. Scale bar 1 µm. **a’** 3D-reconstruction of the interphase Golgi apparatus (of **a**) illustrates its 3D architecture. White lines representing a fraction of MTs, which interlace the Golgi-network. Actin fibers (red), circumjacent the cup-shaped Golgi. See Movie S3. Scale bar 1 µm. **c** Anaglyph image of the Golgi-network in interphase, revealing its three-dimensional architecture by interconnected dictyosomes (D). Several endosomes (E), vesicles (V) and mitochondria (M) are present in between. Scale bar 1 µm. **d** Golgi in early prophase, disintegrating synchronous into stacks and clouds of vesicles and vesicular–tubular clusters. High-resolution reveals that ERGICs (inset) represent the major fraction of the cloud, rather than single vesicles. Scale bar 1 µm; inset 100 nm. **e** Disintegrating Golgi in pro-metaphase: only rudimental dictyosomes (D), formed of vesicular–tubular clusters are present (inset). Scale bar 1 µm; inset 100 nm. **f, g** The Golgi reassembles in telophase. Single, separated dictyosomes form stacks of few cisternae. Typically, they are in direct contact to ER, primary endosome (PE) (**f**) and lipid bodies (**g**). Both, endosomes and lipid bodies have at least one or several connections to the ER. Scale bar 500 nm. **h** Anaglyph image of a dictyosome formed in telophase. Single dictyosomes (D) with characteristic cis- and trans-site are in contact to ER, lipid bodies and endosomes. ERGICs (framed area) are typically observed at the cis-site. Scale bar 1 µm. **i**–**l** Representative 3D-reconstructions of the entire Golgi apparatus (orange), chromatin (purple) and centrosomes (blue, encircled) at different mitotic stages. Scale bars 5 µm. **i** With onset of prophase the Golgi disintegrates rapidly into single dictyosomes, which collapse synchronous into clouds of vesicles and vesicular-tubular clusters. **j** After Golgi disassembly, several small, rudimentary dictyosomes and/or vesicle clusters are present in metaphase. **k** Only few rudimentary dictyosomes and vesicle clusters are still present in anaphase. **l** In telophase, groups of typical dictyosomes, sometimes interconnected, are visible in close proximity to the centrosome (circle) and on the opposite side of the nucleus

In interphase, large dictyosomes are present with distinct cis- and trans-sites, 3-(5) cisternae and abundant peripheral vesicles (Fig. 6a). Dictyosomes cluster at one side of the nucleus in the vicinity of the centrosome forming a network of approx. 30 two-dimensionally interconnected stacks (0.75-3 µm in diameter) (Fig. 6b). The Golgi-network is generally cup-shaped but can form a complex 3D architecture (Fig. 6b; Movie S3). In all interphase cells, some single dictyosomes were randomly distributed. Bundles of actin are present in interphase and all stages of mitosis (Fig. S6). Characteristic is their package of numerous thin electron dense fibrils (Fig. S5), the thinnest with diameters of 6–8 nm. Locally, groups of rather flat electron dense vesicles, aligned in a row, are frequently observed in contact to actin-bundles (Fig. S5b, b’). Sickle-shaped ribbons of actin follow the contour of the Golgi (Fig. 6b; Fig. S5c, c’, d, d’) and numerous microtubules and/or microfilaments interlace the space between the interconnected dictyosomes (Fig. 6a, b). Clouds of vesicles fill the space between the Golgi stacks. Primary endosomes, late endosomes and strands of tubular ER are densely packed (either by attachment or interconnected), within the cloud of dictyosomal vesicles, that a discrimination in 2D is hardly possible (Fig. 6c; Fig. S5c, c’). During prophase, the connections between the dictyosomes and the stacks of disc-shaped cisternae are lost and the entire Golgi disintegrates into clouds of vesicles (Fig. 6d; Fig. S6a, a’), still retaining the 3D organization of the Golgi in interphase (Fig. 6i; compare with Fig. 6b). The vesicle clouds remain in contact to both endoplasmic reticulum (ER) and numerous endosomes (Fig. 6d; Fig. S6a). Strands of actin are randomly distributed between the clouds of vesicles (Fig. S6a, a’). Compared to interphase, the amount of MTs interlacing the Golgi is reduced significantly (Fig. S6a). Volume rendering of 100–150 nm thick layers reveals the vesicles as vesicular–tubular clusters (VTC), or ER-Golgi intermediate compartments (ERGICs) (Fig. 6d, e; Fig. S5a). In pro-metaphase, the disintegration of the Golgi progresses by a reduction of the volume (not number) of the vesicle clouds (Fig. 6e). In metaphase, only a minor fraction of dictyosomal stacks was still present, lacking the typical parallel cisternae (Fig. 6j; Fig. S6b). In anaphase only very few rudimentary dictyosomal stacks were present, scattered in the cytoplasm (Fig. 6k; Fig. S6c); MTs were not observed in their vicinity. In telophase, dictyosomes reappear (Fig. 6–h), the major fraction reassembling in proximity to the centrosome or opposite of the nucleus near the midbody (Fig. 6l). They were not inter-connected, ranging from rudimentary (Fig. 6f, g; Fig. S6d) to mature dictyosomes, with their typical cis- and trans-site (Fig. 6h). After dictyosomal reassembly, VTCs are present at the cis-sites (Fig. 6h). MTs and bundles of actin are visible again, crossing the dictyosomes (Fig. S6d). From telophase to interphase, increased aggregation of dictyosomes forms the characteristic architecture of the Golgi again.

### Interconnected system: ER, endosomes and lipid bodies

The ER forms an extensive network, which consists of sheets and tubules (Fig. 3g, h, a and Fig. S7a). Independent of the stage of the cell cycle, fenestrated sheets are predominantly found in the cell periphery (Fig. 4a). In interphase, tubular ER typically interlaces the bulk of cell organelles located at one side of the nucleus. From pro-meta- to anaphase, ER is typically cap-shaped with approx. 3 parallel layers at both poles (Fig. 4a), significantly reduced from anaphase to telophase to one (if any) polar ER-sheet beneath the plasma membrane. Numerous small VTCs are always distributed within the cytoplasm (Fig. S7b–d). Although variable in size and shape, 3D reconstruction reveals a basic “monomer” structure: short strands of extremely thin, straight or forked tubules, terminated by small vesicles (Fig. S7b–d). Larger aggregates form spider-like discs (Fig. S7c, d). In interphase, although present in large numbers, VTCs are difficult to detect, as they are cryptic between the cell organelles.

**Fig. 7:**
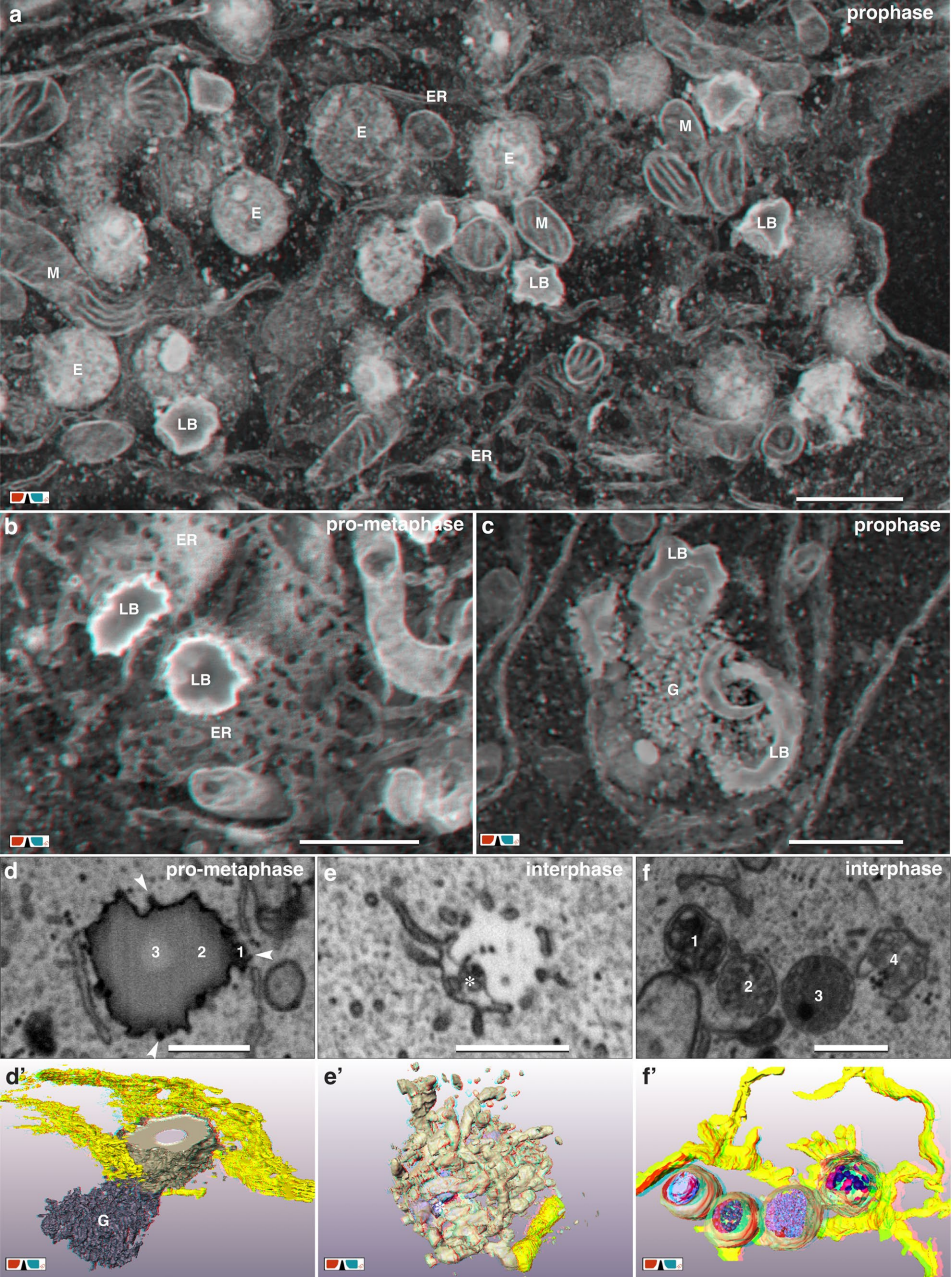
Interconnected system: ER, endosomes and lipid bodies. **a** During all stages of mitosis, endosomes (E), Lipid bodies (LB) and Mitochondria (M) are in compact vicinity to each other and interconnected by ER. (Anaglyph image) Scale bar 1 µm. **b** Lipid bodies, characterized by their electron dense cortex, are circumjacent and in contact with ER-sheets. (Anaglyph image) Scale bar 1 µm. **c** Formation of lipid bodies. Cup-shaped LBs are surrounded by electron dense granular structures (G), which seem to fuse with the cortex of the LBs. (Anaglyph image) Scale bar 1 µm. **d**/**d’** Lipid body (LB; grey), in contact with ER. The LB consists of 3 zones: an electron dense “rough” cortex (1), a less electron dense middle layer (2) and an electron translucent core (3). A cloud of electron dense granules (G) is in direct contact with the LB (d’). Numerous, single granules are in contact with the LB surface (d; arrowheads). Scale bar 500 nm. **e**/**e’** Primary endosome, which is connected to the lumen of the ER (yellow). Vesicles and small tubules start to form an open, hollow sphere, including vesicles and membranous structures (asterisks). Scale bar 500 nm. **f**/**f’** Different types of endosomes, connected to the same strand of ER, containing membranous structures (1), small vesicles (2; 4) or a mixture of vesicles, granules and electron dense inclusions (3). Scale bar 500 nm

Lipid bodies (LB) are prominent structures in all stages of the cell cycle. The number of LBs per cell varies from 30 up to 120. They have a maximal diameter of 1 µm, are irregularly shaped with an uneven electron dense cortex, a middle layer and an inner core (Fig. 7a–d). Nearly all LBs exhibit multiple connections to the ER (Fig. 7a–d) and are in close vicinity to endosomes and dictyosomes (Fig. 7a), implicating a functional relation. Characteristic for a fraction of LBs are electron dense, knob-like protuberances at their surface (Fig. 7d) and an accumulation of small granules or vesicles close to their cortex (Fig. 7c, d’).

Endosomes are abundant (100–250 per cell) in all stages of the cell cycle in HeLa cells (Fig. 7a, e, f). All endosomes are almost spherical, with a diameter ranging between 0.5 and 1.2 µm (Fig. 7a, f). Typically, they are connected to the lumen of the ER (Figs. 6f, 7f) and in contact to dictyosomes (Fig. 6f). Three major types of endosomes can be categorized: (i) primary endosomes = cup-shaped ER, partially filled with vesicles and VTCs connected to their ER-derived membrane (Fig. 7e, e’); (ii) late endosomes = enclosed endosomes including collapsed membranes or vesicles (Fig. 7f, f’); (iii) endosomes with a homogenous, rather electron dense matrix, representing endo-lysosomes (Fig. S6a, a’).

## Discussion

### Metamorphosis of the nuclear envelope

For HeLa cells there are detailed LM investigations on the transition from NE to ER and back to NE (Anderson and Hetzer 2008; Lu et al. 2009). Although the NE disappears as an integral structure in metaphase, large areas of ER-sheets, derived from NE, still outline the shape of the NE (Figs. 3c, 4a) as shown by live cell imaging (Anderson et al. 2009). The NE perforates at the regions facing the centrosomes (Figs. 3b, 4a) in accordance to CLEM data (Domart et al. 2012), allowing MTs access for attachment to kinetochores (Fig. 3b). It is obviously an important strategy of the NE/ER-cage to neatly separate the chromosomes from cytoplasmic constituents during mitosis. Maximum compaction of the chromatin is reached in late anaphase (Mora-Bermúdez et al. 2007), necessary for wrapping of the chromosomes by ER-sheets (Fig. 3d, e, g–h; Movie S2) as shown by LM (Anderson and Hetzer 2008; Anderson et al. 2009; Lu et al. 2009). The center of chromosome bulk is not covered completely due to the uneven chromosome topography and reduced accessibility for ER-sheets (Fig. 3d, e, g, h; Movie S2), resulting in formation of tunnels through the nucleus in telophase, that persist absolutely until interphase (Fig. 3f, i), as shown for numerous organisms (for review see: Malhas et al. 2011).

### Essential role of clamps for midzone formation

**Fig. 8:**
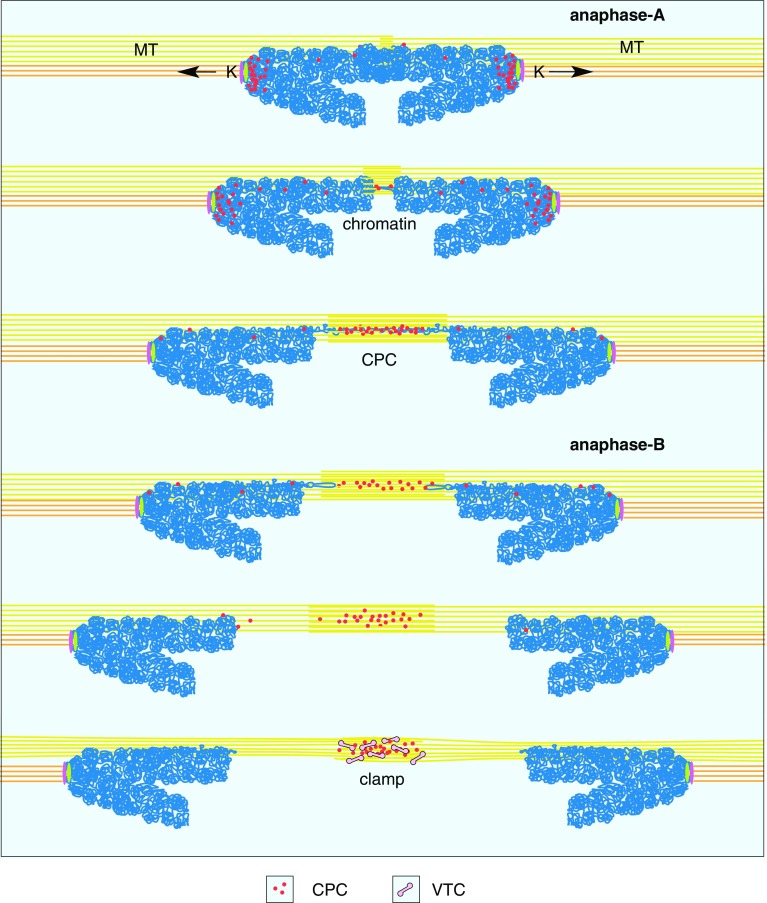
Midzone formation. Midzone formation is mediated by microtubules (MT), passing the arms of separating chromatids and withdrawing chromatin from the telomere regions. Chromosomal passenger complexes (CPC) such as Aurora B, INCENP, borealin and survivin, translocate in anaphase-A from the centromeres to the ends of the chromosomes. Accumulation of CPCs in the midzone is initiated, where antiparallel microtubules overlap. With attachment of VTCs to accumulated CPCs, clamps are formed. Simultaneously, with anaphase progression chromatin strands relocate to their chromosomes, which are pulled to the centrosomes by microtubules, attached to kinetochore (K), whereas clamps remain in the midzone

The paradigm that chromosome segregation is mandatory dependent on MT attached to kinetochores has to be attenuated by observations of kinetochore-independent segregation in *C. elegans*: lateral microtubule–chromosome associations, established during pro-metaphase, remain intact during anaphase to facilitate separation (Muscat et al. 2015). Attachment of MTs to chromosome arms is also typical for HeLa cells, as shown during anaphase (Fig. 4d). Spindle-MTs are attached to the flanks of chromosomes and characteristic electron dense strands, emanating from their telomeric regions (Fig. 4–g). According to structural criteria (electron density, granularity) these strands are simply interpreted as chromatin. DAPI staining may be too weak in intensity to visualize these thin strands within an entire nucleus in presence of condensed bulk chromatin. Further experiments with state of the art super-resolution LM and staining with the most sensitive fluorescent dyes e.g. YOYO-1 could provide specific information already at the LM level during anaphase-A (Pyle and Chen 2017; Rocha et al. 2017). These strands are withdrawn from the chromosomes, which could be explained by pulling forces from the MT-movement. As they are apparently involved in formation of the later midzone, it is hard to believe that a substantial amount of chromatin is spent to build a rather solid structure (midbody), needed for cell fission. An explanation could be, that the translocation of chromosomal passenger complexes (CPC), from the centromeric region along the chromosome arms, is mediated by MTs attached to chromatin, due to their increased microtubule binding affinity (Hümmer and Mayer 2009) (Fig. 8). As chromatin condenses further during anaphase (Mora-Bermúdez et al. 2007), the protuberances are obviously retracted into the chromatin bulk.

Clamp-like structures were already published since the 60th with high quality TEM micrographs, either just named “fibrillar substances” (Buck and Tisdale 1962), “midbody” (Robbins et al. 1968), “CENP-E cross-links” of the interzonal microtubules (Yao et al. 1997), or as an accumulation of dynamin, shown by immuno labeling (Thompson et al. 2002). The characteristic shape of the clamps, 0.5–2 µm long strands, and their orientation in a plane between separating chromosomes (midzone) (Figs. 4f–g, 5a–e; Movie S2), match in size and spindle shape with LM localization of PRC1, MKLP1, KIF4 and CPCs (comprising Aurora B, INCENP, borealin and survivin), as shown by Kurasawa et al. (2004) and presented in detail by publications from the Earnshaw group (for review see: Carmena et al. 2012) (Fig. 8). PRC1 is responsible for cross-linking of overlapping antiparallel microtubules (Schuldt 2010). We, therefore, conclude, according to published data, that the electron dense matrix of the clamps (Fig. 5a) mainly represents microtubule-associated proteins (PRC1), microtubule-based motor proteins (KIF4) and chromosomal passenger complexes (Aurora B, INCENP, borealin and survivin), which bundle and stabilize antiparallel microtubules, thereby forming the midzone (Carmena et al. 2012). 3D-reconstructions reveal that VTCs are attached to the microtubule bundling matrix of the clamps (Fig. 5a), described as “midbody vesicular complexes” in 1969, based on TEM micrographs (Robbins and Jentzsch 1969). The observed clusters of small membranous vesicles located close to the clamps, were assumed to provide a morphological basis for spindle elongation (Robbins and Jentzsch 1969). As it is likely that small vesicular–tubular structures are Golgi derivatives, intermediate compartments (IC) are candidates, representing the vesicles aggregates. This is supported by investigations of NRK cells, providing evidence that the permanent IC elements function as way stations during dispersal of Golgi components at prometa- and metaphase, indicating that they correspond to the Golgi clusters (Marie et al. 2012). Although the IC elements maintain their clustering at the spindle poles during metaphase to telophase, they also associate with the central spindle, imaged with high-resolution CLSM (Marie et al. 2012). As division of plant cells is much different compared to animal cells, especially concerning centrosomes and midbody, there is a fascinating conformance, shown by detailed ultrastructural investigation with electron tomography of meristem cells of *Arabidopsis*, preserved by high-pressure freezing (Seguí-Simarro et al. 2004). The phragmoplast, a homologous structure to the midzone/midbody is formed by accumulation of a vesicle cloud followed by tubulo-vesicular network (TVN) (Seguí-Simarro et al. 2004). Correlative high-resolution CLSM in combination with FIB/SEM could provide important structural information for elucidating the first steps of midzone formation.

## Golgi transition

The Golgi has been studied extensively in structure and function (Sütterlin and Colanzi 2010; Wang and Seemann 2011; Yadav and Linstedt 2011; Gosavi and Gleeson 2017; Wei and Seemann 2017). 3D-EM techniques made data available for the Golgi of plants (Staehelin and Kang 2008) and mammals (Ladinsky et al. 1999; Marsh and Pavelka 2013; Koga et al. 2017). Telophase is most instructive to understand the metamorphosis of the Golgi. The first stacks reappear, distributed in the cytoplasm close to endosomes and lipid bodies, and in contact to ER (Fig. 6f, g). As small clouds of vesicles accompany the first dictyosomes, still reduced in cisternae, their reconstitution from vesicles is likely. Different models exist for Golgi dis- and reassembly during cell division: (i) upon disassembly, Golgi vesicles are completely integrated into the ER, and with onset of telophase, reassemble out of the ER (Zaal et al. 1999; Altan-Bonnet et al. 2006); (ii) ER and the Golgi are considered as independent compartments: the Golgi-network disintegrates into COPI vesicles that are distributed via MTs, and reassembles from these vesicles after mitosis (Jesch and Linstedt 1998; Jokitalo et al. 2001; Seemann et al. 2002; Axelsson and Warren 2004). Within the last two decades it became evident, that the Golgi (beside the centrosome) acts as a microtubule organization center (MTOC) (Chabin-Brion et al. 2001; Nakamura et al. 2012; Zhu and Kaverina 2013; Rios 2014; Sanders and Kaverina 2015; Nishita et al. 2017). Earlier studies have shown that the Golgi reassembly dependents on the actin and microtubule cytoskeleton and their associated molecular motors, which are responsible for transport of vesicular carriers (Brownhill et al. 2009; Tang and Wang 2013), supported by the presented 3D-reconstructions (Fig. 6b; S5; Movie S3).

### Membrane carousel: ER, Golgi, endosomes and lipid bodies

For single-copy cellular elements (chromosomes, centrosomes), a duplication before cell division is essential (Birky 1983). Autonomic mitochondria have to be separated to equip both daughter cells with an adequate population (Birky 1983). All cell constituents present in large numbers or can be synthesized de novo, can be divided in parts of their population (Birky 1983; Lucocq et al. 1987; Lucocq and Warren 1987). The Golgi, predominant in interphase, disintegrates rapidly with onset of pro-metaphase into clouds of vesicles (Fig. 6d, e). In contrast, ER, endosomes and lipid bodies are present all the time during mitosis (Fig. 7). All types of endosomes and endo-lysosomal compartments are directly interconnected with ER (Fig. 7f), forming a common network as shown by light and electron microscopy in Cos-7 cells (Friedman et al. 2013), neurons (Wu et al. 2017) or recently in HeLa cells, were contact sides to early endosomes and late endo-lysosomal compartments are identified with CLEM by (Fermie et al. 2018). There may be principally two different pathways for endosome turnover, summarized by (Huotari and Helenius 2011). A “recycling” pathway characterized by uptake of exogenous material via early endosomes, defined initially as the compartment that first receives incoming cargo and fluid (Helenius et al. 1983) and a cytosolic “degradation” pathway, involving early endosomes, late endosome and lysosomes (for review see: Huotari and Helenius 2011). It is implicated, that the recycling pathway provides the early endosomes, necessary for the degradation pathway. Based on 3D-data we conclude that early endosomes (sensu stricto) are not necessary for an endosome carousel: formation of cup-shaped ER and accumulation/fusion with (dictyosomal) vesicles resulting in a characteristic structure, here defined as “primary endosome” (PE), which is transformed during maturation, further incorporation of vesicles and degradation of vesicle membranes to late endosomes and possibly lysosomes. It is difficult to discriminate between endosomes and lysosomes, as their transition is very dynamic, contradictory to a strict functional separation (Fermie et al. 2018). LAMP-1 is referred as an specific marker for lysosomes (Gowrishankar et al. 2015); however, a recent correlative light and electron microscopy study showed, that LAMP-1-GFP not only correlates with lysosomes, but with all types of endosomes and endo-lysosomal compartments, verified by FIB/SEM microscopy (Fermie et al. 2018). Identifying lysosomes by fluorescence microscopy, using LAMP-1-GFP, may therefore lead to incorrect interpretations concerning lysosomes.

The role of lipid bodies (lipid droplets) has been less clear, as there is only limited ultrastructural information available (Beller et al. 2008; Farese and Walther 2009; Soni et al. 2009; Gao and Goodman 2015). Lipid bodies were long perceived as inert fat particles in animal systems and been largely ignored by cell biologists (Farese and Walther 2009). Interest in the organelle’s cell biology has exponentially increased over the last decade due to the link between LBs and prevalent human diseases (for review see Pol et al. 2014). Typically, LBs are spherical, have a half-unit membrane (Yatsu and Jacks 1972; Martin and Parton 2005), a homogeneous matrix and are formed at the ER in animals (Martin and Parton 2005; Thiam and Beller 2017) and in plant cells at the ER or plastid membranes/envelope (Wanner et al. 1981). ER-sheets enwrapping the LBs is characteristic for both, plants, animals e.g. HeLa cells (Soni et al. 2009), fibroblasts (Martin and Parton 2005), U937 cells (Wan et al. 2007) and Huh7 cells (Fujimoto and Parton 2011). For HeLa cells the contact between LB and ER was quantified, showing that ER-lipid body and ER-endolysosome association is characteristic, deduced from single ultra-thin sections and confirmed with 3D reconstruction with TEM-tomography (Zhao et al. 2017). Typical for LBs is the electron dense cortex, enhanced in contrast by fixation with ferrocyanide-reduced osmium/thiocarbohydrazide/osmium (= rOTO) also shown for HeLa cells by Zhao et al., (2017) and for human mast cells, however, fixed with 2% glutardialdehyde and 1% osmium tetroxide only (Dichlberger et al. 2011). The lipid body cortex is always in contact with ER (Fig. 7a–d) and frequently accompanied by vesicles (Fig. 7c, d). The striking accumulation of Golgi vesicles in prophase, paralleled by vesicle clouds, which are in contact to lipid bodies (Fig. 7a–d), is interpreted as interplay of dictyosomal derivatives with lipid body formation.

The attempt of quantifying lipid bodies for volumetric and for balancing with Golgi membrane and ER volume (provided by the segmentation data), was impeded by the fact, that their number per cell varies widely. Even in dividing cells (telophase), one daughter cell can contain much more lipid bodies compared to the other. When calculating the volume of a Golgi (approx. 36 stacks, each with 3 cisternae) and transforming the membrane area into vesicles (size: 40–80 nm), 360.000 vesicles would be formed. However, compressing the vesicles to the volume of their membrane lipids, the resulting lipid volume would be only 1 µm^3^, fitting into 5 lipid bodies, which is only a minor fraction of those observed (approx. 80/per cell). If the membrane volume of the Golgi disappears rapidly *via* vesicles into the ERELB, significant ultrastructural changes cannot be expected, particularly since the numbers of LBs and endosomes per cell vary widely.

**Fig. 9:**
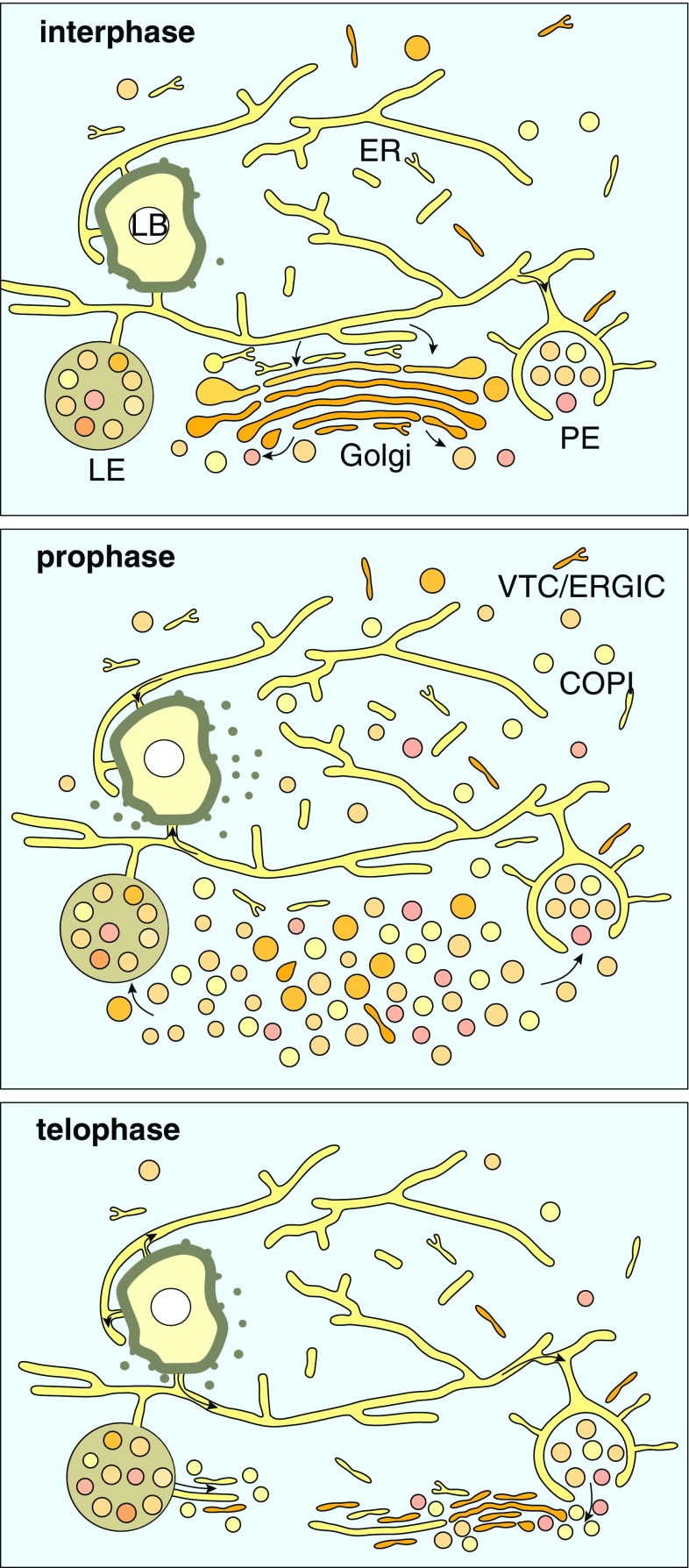
Membrane traffic—the ERELB-network. Elaborate network of ER, endosomes, lipid bodies, Golgi and their mutual relation. All partners are permanently connected *via* endoplasmic reticulum (ER) in interphase, either by fusion with the ER membrane, typical for primary endosomes (PE), which originate from cup-shaped ER-segments, fusing with dictyosomal vesicles or late endosomes (LE), still connected to ER. The lipid bodies are connected by fusion of the outer leaflet of the ER membrane with the half unit membrane of the lipid body. A direct flow of membrane components (arrows) between the compartments is given. During prophase of mitosis the disintegration of the Golgi into vesicles (COPI) or ICs/VTCs/ERGICs and their integration into the ER, primary or late endosomes is achieved by a membrane circulation involving: (i) recycling and distribution by endosomes and (ii) storage of membrane lipids by lipid bodies (LB). During telophase, the Golgi is reassembled in interplay of all partners of the ERELB by reverse flow of membrane components, indicated by arrows

Our data strongly support a formation of endosomes by aggregation of vesicles presumably of dictyosomal origin with direct involvement of ER, forming at least for some time, a luminal connection. As the ER strands are wound around the endosomes like an “umbilical cord”, the luminal connection between ER and endosome matrix will be rarely seen convincingly in ultra-thin sections with TEM. As ER is intimately connected with other organelles, shown by 3D reconstruction (Fig. 7) we postulate that ER, LBs and endosomes play an essential role in membrane turnover of HeLa cells. According to the 3D data, the ER-endosome-lipid body system, here defined as “ERELB”, forms a permanently maintained system, playing the leading role for the turnover of the endomembrane system, discussed since decades in numerous variants based on LM and EM studies (e.g., English and Voeltz 2013; Klumperman and Raposo 2014; Wu et al. 2017). The ERELB-system explains an essential membrane circulation involving: (i) Golgi disintegration and reconstitution; (ii) vesicle/membrane turnover mediated by endosomes; (iii) storage of membrane lipids by LBs (Fig. 9). From the 3D data presented, it is clear that ER, dictyosomes, endosomes and lipid bodies cannot be considered as independent compartments. The categorical separation of structural and functional cellular entities may be responsible for competing opinions; neither model is entirely concurrent with the dynamics of an interconnected system. Whether dictyosomes derive from ER, endosomes and lipid bodies or in a concert of all three partners of the ERELB is still an open question (Fig. 9).

## Capabilities of FIB/SEM

Within the last two decades TEM tomography established as the state of art technique for high-resolution 3D-imaging of resin embedded or cryo-samples. The parameters limiting 3D investigations are section thickness (approx. 300–500 nm) and the small field of view (approx. 5 × 5 µm). For large volumes, especially in combination with 3D LM, three candidates have to be compared: array tomography, 3View^®^ and FIB/SEM. There is no competition between the different techniques, only a decision, which one is most suitable for the scientific question. If several mm^3^ of tissues are needed for 3D-reconstructions, 3View^®^ will be the first, if not only choice. If re-investigations of sections are necessary, only array tomography can achieve this demand. FIB/SEM, with its unsurpassed z-resolution, is recommended for bridging high-resolution LM with TEM tomography. Many cytological questions can be addressed with correlative light microscopy combined with FIB/SEM-tomography of ultra-thin embedded cells in an efficient way.

## Summarizing aspects

Ultra-thin embedding of cells on labeled slides has proven to fulfill all demands for CLEM:

- Thickness of the resin layer can be adapted, as desired, to any specimen by use of an acetone-saturated chamber.
- Immediate and precise correlation between LM and SEM is given.
- Milling plane can be set with high precision.
- Direct access to the target cell makes a ramp needless.
- Milling volume is restricted to the cell volume without any redeposition effects.
- Topography of the target cell is visible during the entire run, allowing immediate corrections on the fly.
- Slides serve as an absolute reference necessary for precise alignment of the FIB-stack.

## Electronic supplementary material

Below is the link to the electronic supplementary material.

Supplementary material 1 (TIF 31389 KB)

Supplementary material 2 (TIF 28416 KB)

Supplementary material 3 (TIF 7420 KB)

Supplementary material 4 (TIF 18440 KB)

Supplementary material 5 (MP4 20170 KB)

Supplementary material 6 (TIF 28893 KB)

Supplementary material 7 (MP4 89160 KB)

Supplementary material 8 (MP4 57030 KB)
