# Supplementary figures and images for: Precise and economic FIB/SEM for CLEM: with 2 nm voxels through mitosis
Source: Histochem Cell Biol. 2018 May 23;150(2):149–70. doi: 10.1007/s00418-018-1681-x (PMC6096567; doi:10.1007/s00418-018-1681-x)

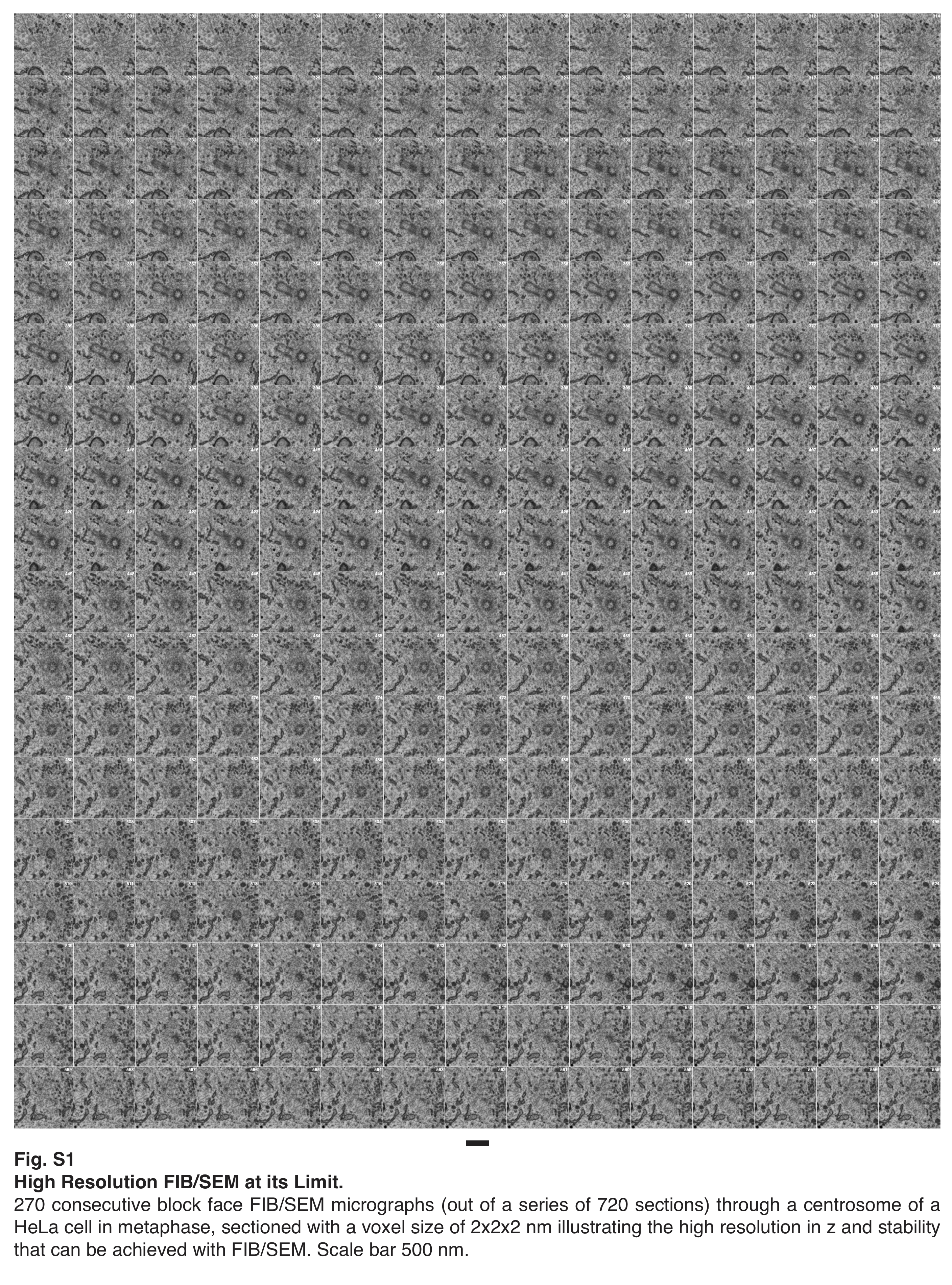

Supplement: Supplementary file 1 — Supplementary material 1 (TIF 31389 KB) [file 418_2018_1681_MOESM1_ESM.tif]

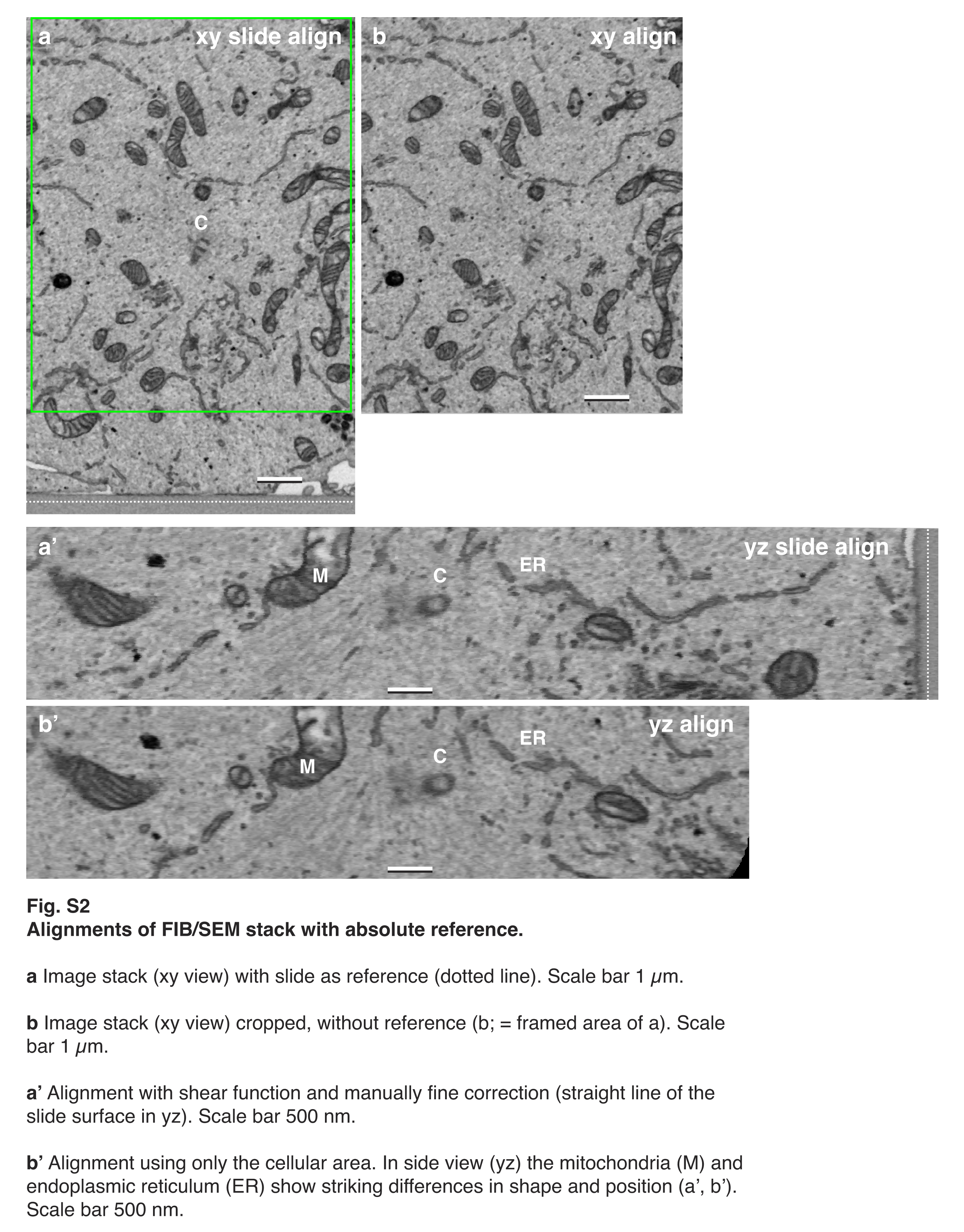

Supplement: Supplementary file 2 — Supplementary material 2 (TIF 28416 KB) [file 418_2018_1681_MOESM2_ESM.tif]

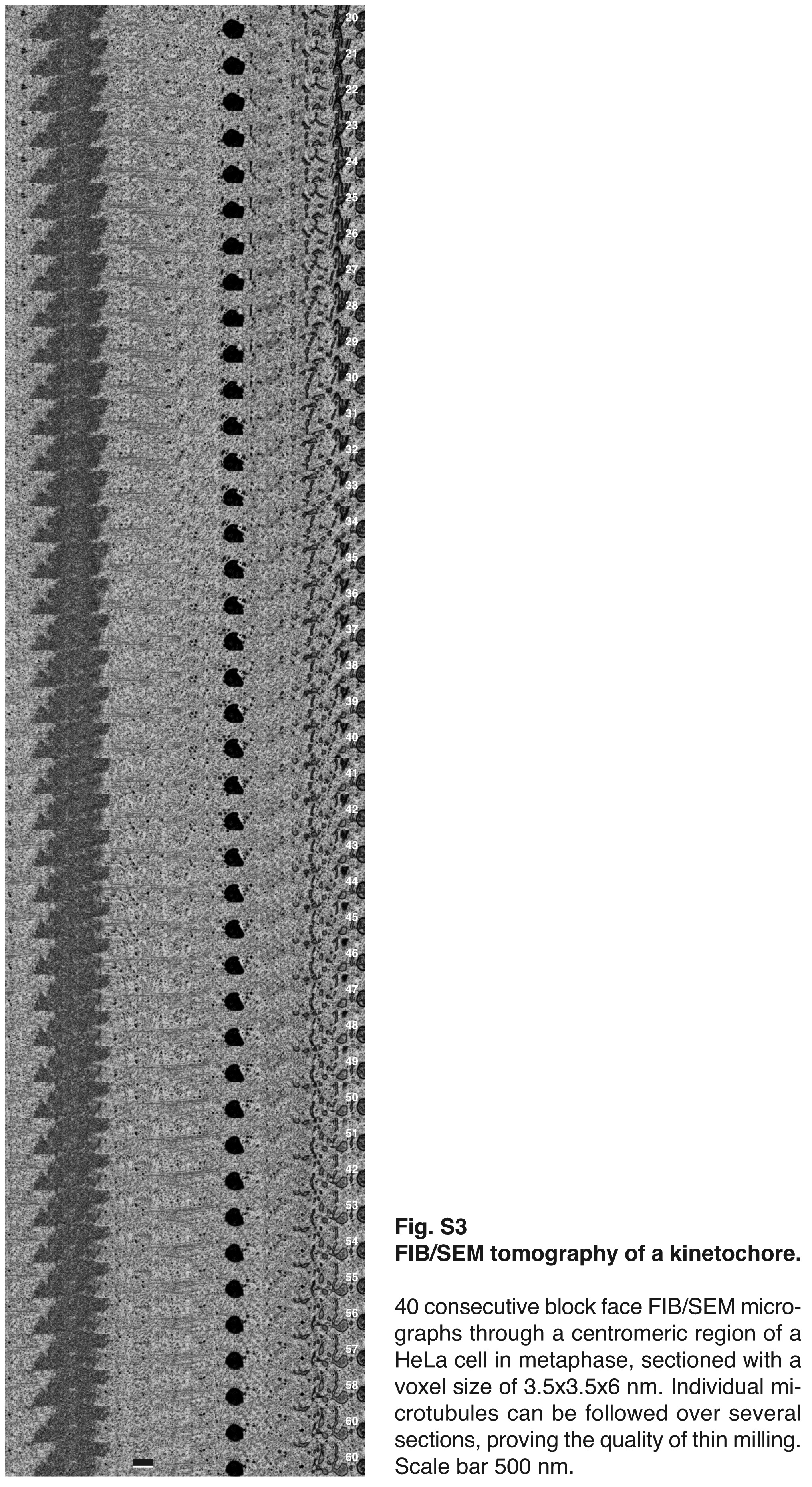

Supplement: Supplementary file 3 — Supplementary material 3 (TIF 7420 KB) [file 418_2018_1681_MOESM3_ESM.tif]

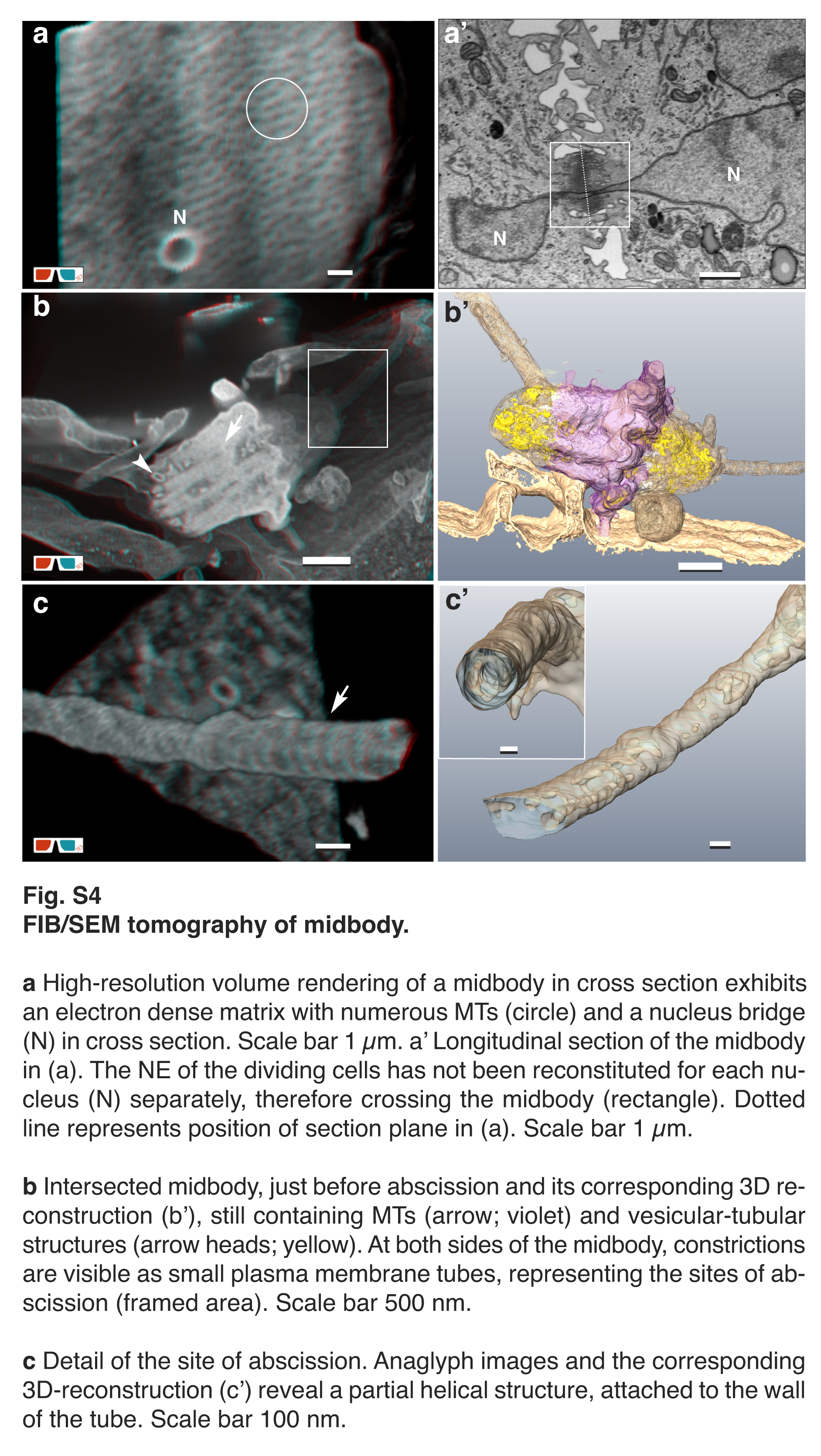

Supplement: Supplementary file 4 — Supplementary material 4 (TIF 18440 KB) [file 418_2018_1681_MOESM4_ESM.tif]

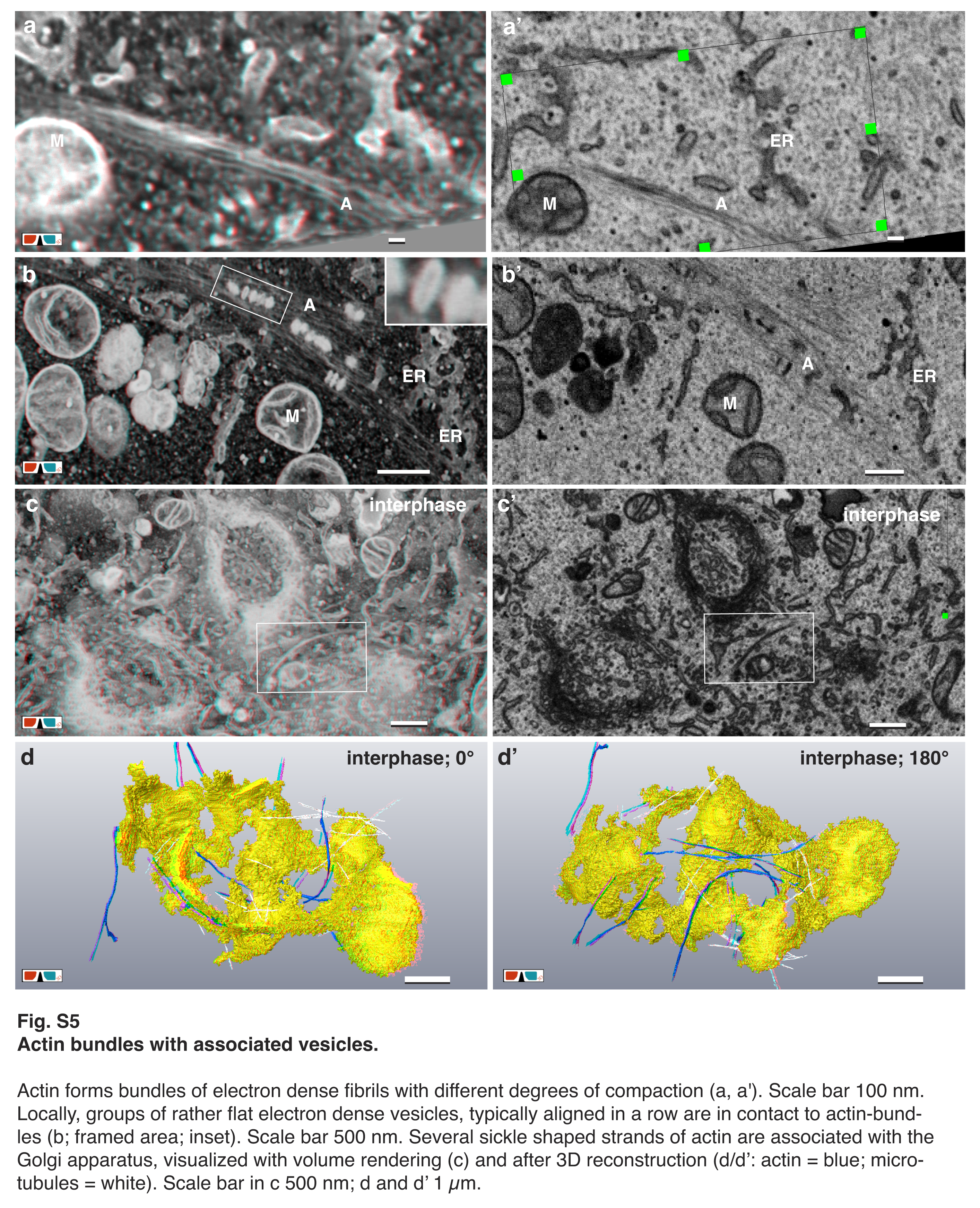

Supplement: Supplementary file 6 — Supplementary material 6 (TIF 28893 KB) [file 418_2018_1681_MOESM6_ESM.tif]
